# The XVIII International Parvovirus Workshop Rimini, Italy, 14–17 June 2022

**DOI:** 10.3390/v15102129

**Published:** 2023-10-20

**Authors:** Giorgio Gallinella, Antonio Marchini

**Affiliations:** 1 Department of Pharmacy and Biotechnology, University of Bologna, 40138 Bologna, Italy; 2 Laboratory of Oncolytic Virus Immuno-Therapeutics, German Cancer Research Centre, 69120 Heidelberg, Germany; a.marchini@dkfz.de

**Keywords:** Parvoviridae, international workshop, conference report

## Abstract

The XVIII International Parvovirus Workshop took place in Rimini, Italy, from 14 to 17 June 2022 as an on-site event, continuing the series of meetings started in 1985 and continuously held every two years. The communications dealt with all aspects of research in the field, from evolution and structure to receptors, from replication to trafficking, from virus–host interactions to clinical and veterinarian virology, including translational issues related to viral vectors, gene therapy and oncolytic parvoviruses. The oral communications were complemented by a poster exhibition available for view and discussion during the whole meeting. The XVIII International Parvovirus Workshop was dedicated to the memory of our dearest colleague Mavis Agbandje-McKenna (1963–2021).

## 1. Introduction

The XVIII International Parvovirus Workshop took place in Rimini, Italy, from 14 to 17 June 2022 as an on-site event—this following a gap in the original schedule in 2020, when the workshop had to be postponed due to the then peaking COVID-19 pandemic.

The Workshop was jointly organized by the Istituto Nazionale Biostrutture e Biosistemi (INBB), Italy, the Department of Pharmacy and Biotechnology and the Rimini Campus of the University of Bologna, Italy (GG), with the support of the German Cancer Research Center, Heidelberg, Germany (DKFZ) (AM).

The decision to organize the meeting as an on-site event allowed us to continue the best tradition of the Parvovirus Workshops, started in 1985 and continuously held every two years, offering the best opportunity to scientists involved in this field of research to meet again, discuss and create networks, in a gratifying venue. About one hundred scientists gathered, contributing to a successful workshop with a total of 64 abstracts.

Following the inaugural session introducing the meeting with three keynote lectures, nine sessions ensued, with communications distributed according to topics, from evolution and structure to receptors, from replication to trafficking, from virus–host interactions to clinical and veterinarian virology, including translational issues related to viral vectors, gene therapy and oncolytic parvoviruses. The oral communications were complemented by a flash poster session, while a poster exhibition remained available for view and discussion during the whole meeting. The abstracts submitted to the conference are reported below as submitted by the authors, in the same order and according to the topics of the actual sessions.

The conference was associated with the Special Issue of *Viruses*, “Advances in Parvovirus Research 2022”, and some of the abstracts can be found as contributing articles. The journal sponsored two awards for young scientists, for the best oral presentation and best poster.

The XVIII International Parvovirus Workshop was dedicated to the memory of our dearest colleague Mavis Agbandje-McKenna (1963–2021). At the end of the inaugural session, Arun Srivastava payed her a deeply affectioned tribute on behalf of the whole community.

## 2. Inaugural Session

In the inaugural session, three keynote speeches were presented. Two of them were reviews on translational aspects in the field. The first, presented by Arun Srivastava, reported on the development of adeno-associated viruses as successful vectors for gene therapy; another, presented by Jürg Nüesch, reported on the potential of a wide portfolio of oncolytic rodent parvoviruses in light of cancer virotherapy. The third speech was presented by Davide Corti, from Humabs BioMed, who offered a wider perspective and reported on the newest techniques and recent applications of human monoclonal antibodies to tackle viral diseases, an issue with the highest relevance to the treatment of severe infections in clinical settings, including parvoviruses.

### 2.1. Keynote 1: AAV: From Almost a Virus to an Awesome Vector (Or Is It?)

*Arun Srivastava*

To date, recombinant adeno-associated virus (AAV) vectors, based on a non-pathogenic parvovirus, have been, or are currently in use, in 284 Phase I/II/III clinical trials for a wide variety of human diseases. In some cases, such as Leber’s congenital amaurosis, lipoprotein lipase deficiency, hemophilia B, aromatic L-amino acid decarboxylase deficiency, choroideremia, Leber hereditary optic neuropathy, hemophilia A and spinal muscular atrophy, unexpected, remarkable clinical efficacy has also been achieved. Several AAV serotype vectors are available at present, which have shown clinical efficacy in a number of human diseases in animal models. To date, two AAV “drugs”—Luxturna and Zolgensma—have been approved by the Food and Drug Administration (FDA). Despite these remarkable achievements, it has become increasingly clear that the first generation of AAV vectors currently in use are not optimal. For example, despite their efficacy in animal models, these vectors have failed to show clinical efficacy in some cases. In addition, relatively large vector doses are needed to achieve clinical efficacy. The use of high doses has been shown to provoke host immune responses culminating in serious adverse events, and more recently, in the deaths of six patients. Thus, it has become increasingly clear that there is an urgent need for the development of the next generation of AAV vectors that are: (i) safe; (ii) effective; and (iii) human-tropic. Since AAV evolved as a virus, and not as a vector for the purposes of delivery of therapeutic genes, the host immune system cannot distinguish between AAV as a virus versus AAV as a vector. Thus, the use of AAV vectors composed of naturally occurring capsids is likely to induce immune responses, especially at high doses since the host immune response is directly correlated to the AAV vector dose. Similarly, AAV as a virus does not express its own genes effectively since its single-stranded DNA genome is transcriptionally inactive. Most of the ssAAV vectors currently in use are also sub-optimal in expressing therapeutic genes. And finally, the tropisms of AAV vectors in animal models do not necessarily translate well in humans, and hence the need to identify and further develop human-tropic AAV vectors. In this presentation, strategies to overcome each of the limitations of the first generation of AAV vectors will be presented. The next generation of AAV serotype vectors promise to be: (i) efficacious at significant reduced doses; (ii) likely to be less immunogenic, thereby increasing the safety; (iii) obviating the need for the use of immune suppression; (iv) reducing vector production costs; and (v) ensuring translation to the clinic with a higher probability of success, for gene therapy of a wide variety of human diseases.

### 2.2. Keynote 2: Tackling Viral Infections by Human Monoclonal Antibodies

*Davide Corti*

Monoclonal antibodies (mAbs) have revolutionized the treatment of several human diseases, including cancer, autoimmunity and inflammatory conditions, and represent a new frontier for the treatment of infectious diseases. In the last twenty years, innovative methods have allowed the rapid isolation of antibodies from convalescent subjects, humanized mice or in vitro libraries, and have proven that the swift isolation of mAbs is a rapid and effective countermeasure to emerging pathogens. During the past two years, an unprecedented wave of monoclonal antibodies has been developed to fight COVID-19. We provide an overview of the preclinical and clinical development of monoclonal antibodies against Ebola virus, SARS-CoV-2, Influenza A virus and hepatitis B virus, including their antiviral and immunological mechanisms of action.

### 2.3. Keynote 3: Generating a Portfolio of Oncolytic Rodent Parvoviruses

*Jürg P.F. Nüesch*

Although the initial H-1PV treatment of cancer patients alone, or in combination with approved anticancer agents, provided promising results, there are still significant limitations. In particular, there were patients that did not respond to the therapy and there are cancer entities, which appear to be of limited targets for H-1PV infection. To tackle these drawbacks, we intend to generate a portfolio of oncolytic parvoviruses, based on additional PV strains that were, like H-1PV, originally isolated as an opportunistic infectant of human tumor cell lines. In addition, we intend to improve the propagation and spreading capacity of H-1PV and other isolates through adaptation towards refractory cancer entities. Indeed, the serial adaptation of H-1PV proved to improve the replication and propagation capacity of the original isolate not only for the target human GBM cell lines, but also for a variety of other cancer entities, such as malignant melanoma and PDAC.

In order to enlarge the repertoire of oncolytic PVs, we first assessed viral sequences in the replicative-form DNAs of a number of virus isolates collected mainly in the late 1960s–early 1970s and a few “younger” virus preparations. These sequences founded the basis to generate replication-competent plasmids, essential tools to produce individual virus entities and to characterize them for their propagation capacity in candidate human cancer cell lines. To date, we obtained a number of replication-competent viruses, derived from H-1, KRV, H3, X14, TVX, LuIII and MVM, and generated (specific) monoclonal antibodies enabling us to determine their properties in various target cells. At present, we use these individual viruses to determine their host range and oncosuppressive capacity, targeting human tumor cell lines derived from brain tumors, malignant melanomas, colon cancer and hepato-cell carcinomas.

## 3. Session 1: Evolution and Structure

### 3.1. Review Lecture: Evolution of Canine and Feline Parvoviruses, and the Control of Viral Host Range and Antibody Neutralization

*Colin R. Parrish, Ian Voorhees, Heather Callaway, Hyunwook Le *and* Susan Hafenstein*

Canine parvovirus (CPV) emerged during the mid-1970s, spread globally during 1978 and has maintained a pandemic circulation in dogs. We examined the whole-genome evolution of the virus since it emerged as a variant of a feline-like virus and found that CPV was able to traverse species barriers and rapidly adapt to new hosts, both in nature and in experimental studies. However, only a small number of nucleotide substitutions (fewer than 40 in total) became fixed or widespread throughout the entire evolution of this virus, with parallel evolution and reversion occurring on multiple occasions. Of the substitutions that are fixed or widespread, synonymous changes make up most within the viral non-structural gene (NS1), while most within VP2 are nonsynonymous and many influence host cell receptor binding and infection, mapping within or close to the footprint of the transferrin receptor or antibody binding sites.

We also examined capsid functions and show that the substitutions of residues on the exterior capsid surface altered the binding affinity between the capsid and the transferrin receptor type 1 (TfR). Some altered host range and others allowed uptake into cells without infection, showing that specific interactions between the TfR and the capsid are required for infection. Some of the same mutations also altered antibody binding to the capsid, confirming that they changed the local surface structure. Other changes include a reduction in viral infectivity by 1000-fold or more involved residues inside the capsid structure. These included two cysteine residues and the structures surrounding them. One of these mutations, Cys270Ser, altered capsid stability and influenced a VP2 cleavage that occurs in ~10% of the major capsid proteins. These results confirm the complex nature of the parvovirus capsid and the process of infection of cells.

### 3.2. Comparative Analysis Reveals the Long-Term Co-Evolutionary History of Parvoviruses and Vertebrates

*Matthew A. Campbell, Shannon Loncar, Robert Kotin *and* Robert J. Gifford*

Parvoviruses (family *Parvoviridae*) are small DNA viruses that cause numerous diseases of medical, veterinary and agricultural significance and have important applications in gene and anticancer therapy. DNA sequences derived from ancient parvoviruses are common in animal genomes and the analysis of these endogenous parvoviral elements (EPVs) has demonstrated that the family, which includes twelve vertebrate-specific genera, arose in the distant evolutionary past. To date, however, such ‘paleovirological’ analysis has only provided glimpses into the biology of parvoviruses and their long-term evolutionary interactions with hosts. In this paper, we comprehensively map EPV diversity in 752 published vertebrate genomes, revealing the defining aspects of ecology and evolution within individual parvovirus genera. We identified 364 distinct EPV sequences and show that these represent ~200 unique germline incorporation events, involving at least five distinct parvovirus genera, which took place at points throughout the Cenozoic Era. We used the spatiotemporal and host range calibrations provided by these sequences to infer defining aspects of long-term evolution within individual parvovirus genera, including mammalian vicariance for the genus *Protoparvovirus* and inter-class transmission for the genus *Dependoparvovirus*. Moreover, our findings support a model of virus evolution in which the long-term co-circulation of multiple parvovirus genera in vertebrates reflects the adaptation of each viral genus to fill a distinct ecological niche. Our discovery that parvovirus diversity can be understood in terms of genus-specific adaptations acquired over millions of years has important implications for their development as therapeutic tools—we show that these endeavors can be approached from a rational foundation based on comparative evolutionary analysis. To support this, we published our data in the form of an open, extensible and cross-platform database designed to facilitate the wider utilization of evolution-related domain knowledge in parvovirus research.

### 3.3. Odegus 4 Is an Endogenous Parvoviral Element with Antiviral Function

*Angelica Bravo, Leandro Fernandez, Rodrigo Ibarra-Karmy, Robert J. Gifford *and* Gloria Arriagada*

Endogenous viral elements (EVEs) are viral-derived DNA sequences present in the genome of extant species, where an endogenous parvoviral element (EPV) represents an ancestral parvoviral infection in a host germ line. Some of their EVEs possess open reading frames (ORFs) that can express proteins with physiological roles in their host. Furthermore, it has been described that some EVEs exhibit a protective role against exogenous viral infection in their host, but no EPV has been to date associated with this function. Previously, our laboratory demonstrated that an EPV is transcribed in the liver of *Octodon degus* (degu). This EPV, named Odegus4, contains an intact ORF that possess the Rep protein domain of depend-parvovirus. We also demonstrated that, in cell lines transfected with a plasmid that encodes Odegus4, it has nuclear localization. These characteristics lead us to propose that Odegus4 may function as a cellular coopted protein in degu. 

We assess whether Odegus4 is protein with an antiviral role against exogenous parvovirus. We cloned the 1527bp sequence of OD4 into bacterial and eukaryotic expression vectors. Using bacterially produced proteins, we obtained polyclonal antibodies that specifically recognize OD4 over other EPVs in Western blot. Using these antibodies, we detected the presence of OD4 protein in degu tissues. To test if Odegus4 could protect against exogenous parvovirus, the minute virus of mice (MVM), a model of autonomous parvovirus, was generated in cells expressing Odegus4 or the control, resulting in a less effective viral infectivity when Odegus4 was present. We also consider if, in cells, the expression of Odegus4 MVM infection was impaired. For this, we transfected cells with Odegus4-encoding plasmids or an empty vector, synchronized them and infected them with MVM. We observed that MVM protein expression, DNA damage induced by replication, viral DNA and cytopathic effect were reduced when Odegus4 was present. Our results show that Odegus4 is expressed as a protein in its host and show, for the first time, that an EPV has a protective role against the extant parvovirus.

### 3.4. Capsid Structures of Aleutian Mink Disease Virus and Human Parvovirus 4: Adding New Faces to the Family Portrait

*Mario Mietzsch, Renuk Lakshmanan, Alberto Jimenez Ybargollin, Paul Chipman, Xiaofeng Fu, Jianming Qiu, Maria Söderlund-Venermo, Robert McKenna *and* Mavis Agbandje-McKenna*

Parvoviruses are ssDNA viruses with small, non-enveloped capsids with T = 1 icosahedral symmetry. Determining the capsid structures provides a framework to annotate regions important to the viral life cycle. However, to date, the capsid structures have been determined for only four of the ten *Parvovirinae* genera. Aleutian mink disease virus (AMDV), a pathogen of minks, and human parvovirus 4 (PARV4) infecting humans are parvoviruses belonging to the genera *Amdoparvovirus* and *Tetraparvovirus*, respectively. While Aleutian mink disease caused by AMDV is a major threat to mink farming, resulting in significant economic losses, no clear clinical manifestations have been established following an infection with PARV4 in humans. For both genera *Amdoparvovirus* and *Tetraparvovirus*, no capsid structures have been determined to date. We present the capsid structures of AMDV and PARV4 determined by cryo-electron microscopy and 3D image reconstruction with ~3 Å resolution. Despite the low amino acid sequence identity (10–30%) with parvoviruses of other genera, both viruses share common features previously observed with other parvoviruses, such as depressions at the icosahedral 2-fold axes and surrounding the 5-fold symmetry axes, protrusions surrounding the 3-fold axes and a channel at the 5-fold axes. However, both AMDV and PARV4 display major structural variabilities in the surface loops when the capsid structures are superposed onto other parvoviruses. Of note, AMDV possesses the largest major viral protein (VP) with 647 amino acids compared to known parvovirus capsid structures (including PARV4), in the range of ~530–590 amino acids. These additional amino acids may compensate for AMDV’s very short VP1u region, which also lacks a phospholipase A2 (PLA_2_) domain and results in large insertions in the majority of the surface loops of the capsid. The capsid structures of AMDV and PARV4 will add to our current knowledge of the structural platform for parvoviruses and permit the future functional annotation of these pathogens, which will help to understand their disease mechanisms at a molecular level towards the development of therapeutics.

## 4. Session 2: Receptors

### 4.1. Receptor and Antibody Interactions with AAV by Cryo-EM/ET

*Guiqing Hu, Mark Silveria, Edward Large, Nancy Meyer, Grant Zane, Scott Stagg *and* Michael S. Chapman*

Most AAV serotypes are dependent on the transmembrane AAV Receptor (AAVR) for endosomal cell entry. Cryo-electron microscopy (cryo-EM) at up to a 2.5 Å resolution reveals interactions of several serotypes with the most strongly bound domain. Intriguingly, AAV5 and the related AAVGo.1 bind PKD1 at a site that is different from that where AAV2 and other viruses interact with PKD2. The epitopes of a dozen monoclonal antibodies have previously been mapped on several serotypes by cryo-EM. The mechanisms of neutralization were sometimes thought to involve interference with glycan attachment or a post-entry step. There is, however, a stronger spatial correlation between neutralizing epitopes and the respective AAVR binding sites than what would be expected if neutralization was conducted by the inhibition of AAVR binding. Upon the re-examination of an exemplary AAV5 antibody, competitive binding inhibition was confirmed in vitro. Although only single domains of AAVR were seen in the high-resolution structures of the complexes, PKD1 has an accessory role (to PKD2) in the interactions of AAV2. The complexes of AAV2 and AAV5 with two-domain fragments of AAVR (PKD12) were examined by cryo-electron tomography (ET) to locate the domains “missing” at a high resolution. Each is in several different configurations extending away without significant interactions at the AAV surface.

Cryo-EM maps of AAV5-PKD12 complexes are low-pass-filtered to reveal a less-ordered structure, including strands of polypeptide adjacent to PKD1 near the AAV5 surface. Side chains are insufficiently ordered for sequence identification, but it is plausible that the peptide is a fragment of either another AAVR subunit or part of the N-terminal region of the AAV capsid protein. The cryo-ET of the AAV2-PKD12 complex shows partially ordered features on the inner surface near the 2-fold axis. Corresponding features have previously been interpreted as the N-terminal region of capsid proteins. The feature is present when βA is weak in high-resolution cryo-EM maps, and this varies among samples of AAV2 complexed with AAVR, but not AAV5. There appears to be a finely balanced conformational equilibrium, perhaps explaining why it has been difficult to rationalize such features in past structures.

### 4.2. Characterization of Glycan Binding by AAV44.9, a Member of the New AAV Clade

*G. Di Pasquale, S. Afione, M. Khalaj, C. Zheng, B. Grewe *and* J.A. Chiorini*

The recent isolation of novel AAV serotypes has led to significant advances in our understanding of parvovirus biology and vector development for gene therapy by identifying vectors with unique cell tropism and increased efficiency of gene transfer to target cells. AAV44.9 is a natural isolate originally found as a contaminate of the laboratory stock of SV15 adenovirus. Its sequence homology places it between current clades in a small cluster with Rh.8R. Recent studies have suggested that AAV44.9 is a promising candidate for photoreceptor-targeted gene therapies and other tissues. To better understand its activity, we used glycan arrays and competition experiments to define its binding as primarily interacting with glucose or glucosamine on N-linked cell-surface carbohydrates. Mutagenesis experiments were conducted to identified the amino acids responsible for glycan recognition and specificity.

### 4.3. Human Parvovirus B19 Infection: A Tale of Two Receptors

*Jan Bieri, Cornelia Bircher, Ruben Assaraf, Remo Leisi *and* Carlos Ros*

Parvovirus B19 (B19V) is a human pathogen with a marked tropism for erythroid progenitor cells (EPCs) in the bone marrow. The N-terminal of the VP1 unique region (VP1u) of B19V and related non-human primate erythroparvoviruses contains a receptor-binding domain (RBD). The VP1u RBD mediates virus uptake in EPCs through interaction with an as-yet-unknown receptor, VP1uR. By using a phage-RBD construct, the expression profile of VP1uR was quantitatively analyzed in multiple cell types. The results confirm the exclusive association of VP1uR with cells of erythroid lineage. In contrast to VP1uR, globoside is ubiquitously expressed, and although it is not required for virus uptake, it has an essential role at a post-entry step by facilitating endosomal escape. We found that pH modulates the affinity between the two receptors. At an acidic pH, the affinity for VP1uR decreases and increases for globoside, which allows for the interaction to occur in acidic endosomes. The finding that B19V affinity for VP1uR and globoside is tightly controlled by the pH has major consequences on the overall virus infection. Under the neutral pH conditions of the extracellular milieu, B19V does not interact with globoside. This strategy prevents the redirection of the virus to nonpermissive cells, promoting the selective targeting of the EPCs. However, considering the ubiquitous expression of Gb4, naturally occurring acidic niches become potential targets for the virus.

### 4.4. Globoside and the Mucosal pH Mediate Parvovirus B19 Entry through the Airway Epithelium

*Corinne Suter, Minela Colakovic, Jan Bieri, Mitra Gultom, Ronald Dijkman *and* Carlos Ros*

The mechanism of entry of parvovirus B19 (B19V) via the respiratory route is unknown. B19V targets the VP1u cognate receptor expressed exclusively on permissive erythroid progenitors in the bone marrow. Following uptake, B19V shifts the receptor in the acidic endosomes and targets globoside, which is required for infectious trafficking. The pH-dependent interaction with globoside may allow virus entry through the naturally acidic nasal mucosa. To test this hypothesis, human airway epithelial cells (hAECs) and polarized monolayers of MDCK II cells were grown on transwell membranes and used as barrier models to study the interaction and transport of viral particles across the airway epithelium. Globoside expression was detected in the ciliated population of hAECs and, under the acidic conditions of the nasal mucosa, B19V was transported to the basolateral side by transcytosis without signs of productive infection. Similarly, MDCK II cells express globoside and support virus transcytosis in a time- and pH-dependent manner. Neither virus attachment nor transcytosis were observed in wild-type cells under neutral pH conditions or in globoside knockout MDCK II cells, demonstrating the direct role of globoside and an acidic pH in the process. Globoside-dependent virus uptake occurred by clathrin-independent, dynamin-, cholesterol- and caveolae-dependent mechanisms and was inhibited by specific antibodies derived from infected individuals. This study provides the first mechanistic insight into the transmission of B19V through the respiratory route and reveals novel vulnerability factors of the airway barrier to viruses.

### 4.5. Viral Capsid, Antibody and Receptor Interactions: Understanding the Binding, Neutralization, Antibody Escape and Receptor Binding Sites of Canine Parvovirus

Robert A. López-Astacio, Daniel J. Goetschius, Hyunwook Lee, Wendy S. Weichert, Oluwafemi Adu, Brynn K. Alford, Ian E.H. Voorhees, Sarah Saddoris, Laura B. Goodman, Edward C. Holmes, Susan L. Hafenstein *and* Colin R. Parrish*

Viruses circulating in nature evolve and frequently emerge as new variants due to evolutionary forces that can result from mutation and selection—including key interactions with receptors and antibodies. We study the evolution of canine parvovirus (CPV) incubated with neutralizing antibodies to reveal how they are controlled by the dynamics of binding, neutralization, antibody escape and overlap with the receptor binding site. CPV is a non-enveloped virus with a single-stranded DNA genome, and it causes serious disease in animals worldwide. The original strain (CPV-2) emerged as a new dog pathogen during the late 1970s due to a host jumping event. The subsequent natural evolution of the virus in dogs and other hosts has altered both receptor and antibody binding, with some single changes affecting both functions. Our study reveals that only a small number of mutations arise within the viral capsid (VP2) under the in vitro selection with each of two neutralizing antibodies, which bind distinct sites on the capsid and show the different levels of overlap with the transferrin receptor (TfR) attachment site. Mutations occurred primarily within the antibody footprints, and few changes occurred in the TfR-binding footprint. Remarkably, 54% of the antibody-selected mutations identified were also present in natural circulating variants, showing the connections between our in vitro study and natural infection and also the potential for the emergence of variants in nature due the host humoral immune response. To understand escape mutation dynamics, we also engineered the capsid-binding sites of the two antibodies. The in vitro selection experiments with the engineered antibodies also showed similar mutations to those selected by the wild-type, despite the alternative interactions with the capsid. This study suggests the potential mechanisms by which these variants emerged in nature and provides a better understanding of the coordinated interactions between antibodies and receptors.

## 5. Session 3: Replication

### 5.1. Cryo-EM Structure of the Rep68-AAVS1 Complex

*R. Jaiswal, V. Santosh *and* C.R. Escalante*

Adeno-associated virus (AAV) is the only known eukaryotic virus with the property of establishing latency by integrating site-specifically into the human genome. The integration site known as AAVS1 is located in chromosome 19 and contains multiple GCTC repeats that are recognized by the AAV non-structural Rep proteins. These proteins are multifunctional, with an N-terminal origin-binding domain (OBD) and a helicase domain (HD) joined together by a short linker. In a previous work using analytical ultracentrifugation, we showed that Rep68 binds to AAVS1, forming a heptameric complex. We report the cryo-EM structures of Rep68 bound to a 50 bp AAVS1 fragment in the apo and ATPγS states. The structure shows that, indeed, Rep68 forms a heptameric ring encircling the DNA. In the apo state, most of the contacts are made by the small oligomerization domain. In addition, three of the seven OBDs remain engaged to the RBS sites. Of the remaining four OBDs, we observed three OBDs forming a dimer with each of the DNA-bound OBDs. Upon binding ATPγS, the helicase heptameric ring closes upon the DNA, where three HDs make backbone DNA interactions. We hypothesize that cycles of ATP hydrolysis and the opening and closing of the helicase ring may cause enough distortions to induce the melting of DNA. 

### 5.2. Insights into the ITR Melting Mechanism: Cryo-EM Structure of the Rep68–ITR Complex

*R. Jaiswal, V. Santosh, A. Washington, B. Braud *and* C.R. Escalante*

The AAV origin of replication consist of three motifs, the Rep binding site (RBS), the terminal resolution site (TRS) and the hairpin Rep binding element (RBE’). During DNA replication, AAV Rep proteins bind to the RBS to perform a site- and strand-specific endonuclease reaction at the TRS that is required to complete the DNA replication of the 3′ hairpin end. A pre-requisite of the nicking reaction is a DNA-melting step using the motor activity of the Rep proteins. To understand the molecular events leading to the melting of DNA, we determined the structure of Rep68 in a complex with AAV-2 ITR. The structure shows that Rep68 forms a heptameric complex in a similar way to the Rep68–AAVS1 complex. The ITR molecule acquires a conformation where two of the arms stacked coaxially and the RBE’ arm protrudes in an antiparallel orientation. The complex shows that one of the OBD molecules interacts with both the RBE’ tip and the RBS at the same time. The structure also shows several residues in the helicase domain that are critical for DNA melting. Taken together, our structure provides insights into how Rep proteins melt the ITRs.

### 5.3. AAV2 Can Replicate Its DNA by a Rolling Hairpin or Rolling Circle Mechanism, Depending on the Helper Virus

*Anouk Lkharrazi, Kurt Tobler, Anita Meie, Bernd Vogt *and* Cornel Fraefel*

Adeno-associated virus type 2 (AAV2) is a small, non-pathogenic, helper virus-dependent parvovirus with a single-stranded (ss) DNA genome of approximately 4.7 kb. AAV2 DNA replication requires the presence of a helper virus, such as adenovirus type 5 (AdV5) or herpes simplex virus type 1 (HSV-1), and is generally assumed to occur as a strand-displacement rolling hairpin (RHR) mechanism initiated at the AAV2 3′ inverted terminal repeat (ITR) end. We recently showed that AAV2 replication supported by HSV-1 leads to the formation of double-stranded head-to-tail concatemers, which provides evidence for a rolling circle replication (RCR) mechanism (Meier et al., 2021; doi: 10.1371/journal.ppat.10096389). Using Southern analysis and high-throughput sequencing (Nanopore and PacBio sequencing), we revisit AAV2 DNA replication and specifically compare the formation of AAV2 replication intermediates in the presence of either HSV-1 or AdV5 as the helper virus. The results confirm that the AAV2 DNA replication mechanism is helper-virus-dependent and follows a strand-displacement RHR mechanism, when AdV5 is the helper virus, and primarily an RCR mechanism when HSV-1 is the helper virus. In addition to the monomeric and multimeric products of the AAV2 genome, a considerable fraction of ITR repeats was observed, particularly in the presence of AdV5.

### 5.4. Consequence and Mechanism of Chk1 Inactivation by MVM during Its Infection

*Igor Etingov and David Pintel*

We and others have shown that minute virus of mice (MVM) infection causes significant damage to cellular DNA and is accompanied by, and exploits, a DNA damage response (DDR). Viral replication centers are localized in the vicinity of cellular DNA damage sites, which attract numerous factors required for viral expression and replication. The infection-induced DDR is transduced by the ATM kinase, while the ATR kinase is inactivated. During infection, the major cell cycle regulator kinase Chk1, a target of ATR, is not phosphorylated at position S345, which inhibits the activation of its G2/M checkpoint function, despite an ongoing DDR and the presence of RPA-coated viral single-stranded DNA. Chk1 is also involved in additional cellular processes, including the homologous recombination repair (HRR) of DNA, and the phosphorylation of the essential HRR factor RAD51. 

We investigate the significance of Chk1 inactivation for MVM replication as well as the mechanism involved in the inhibition of Chk1 phosphorylation upon infection. Using comet assays to assess cellular DNA damage levels and fluorescent reporter constructs to estimate HRR efficiency upon the infection, combined with the overexpression of constitutively active Chk1, we demonstrate that the MVM-induced inactivation of Chk1 significantly reduced the cellular ability to repair its DNA, which facilitates increased levels of viral DNA replication. ATR is known to be recruited to RPA-covered single-stranded DNA fragments in assemblage with its auxiliary factors TopBP1 and the Rad9–Rad1–Hus1 (9-1-1) complex, which are also required for ATR activation and its ability to phosphorylate Chk1. The overexpression of the TopBP1 ATR activation domain (AAD) successfully activated ATR upon infection, demonstrating that MVM incapacitates TopBP1′s ability to support ATR functionality. TopBP1 is known to associate with Rad9, and the phosphorylation of Rad9 at S387 is needed for its capacity to activate ATR. We show that MVM NS1 significantly reduced this phosphorylation, thus identifying a critical role in the prevention of phosphorylation (and thus activation) of Chk1 at S353. The known NS1 association with the casein kinase II α (CKIIα) domain and the possible recruitment of phosphatase PP2C by the NS1–CKIIα complex appeared to be required for the inhibition of Rad9 S387 phosphorylation.

### 5.5. The Autonomous Parvovirus Minute Virus of Mice Localizes to the Cellular Sites of DNA Damage Using Its Non-Structural Protein NS1

*Lauren Bunke, MegAnn Haubold, Clairine Larsen, Rhiannon Abrahams, Sarah Rubin, Isabella Jones, Jessica Pita Aquino *and* Kinjal Majumder*

Minute virus of mice (MVM) is an autonomously replicating parvovirus that is lytic in murine cells and transformed human cells, causing a pre-mitotic cell cycle arrest during which the virus replicates. MVM replicates in nuclear replication centers termed autonomous parvovirus-associated replication (APAR) bodies, rich in factors necessary for virus replication and expression, as well as multiple DNA damage response (DDR) proteins. MVM infection induces ATM-dependent and ATR-independent cellular DDRs. Strikingly, super-resolution imaging and chromatin immunoprecipitation (ChIP) assays revealed that only a subset of cellular DDR proteins, such as MRE11, associates directly with the MVM genome, whereas downstream DDR markers, such as gamma H2AX, surround APAR bodies, likely marking damaged cellular genomic sites. Using chromosome conformation capture assays in trans, we identified the cellular sites of direct interaction between the linear MVM genome and the cellular chromosome, demonstrating that, rather than recruiting DDR proteins to APAR bodies de novo, MVM genomes localize to genomic sites that are abundant in DDR proteins. Some of these cellular DDR sites are fragile sites where oncogenic viruses, such as HPV, localize. These genomic regions are packaged into spatially distinct topologically associating domains (TADs), which also colocalize with accessible Type A chromatin. 

Ectopically expressed MVM-NS1 associates with cellular DDR sites at fragile genomic regions. Interestingly, NS1 bound to the MVM genome is essential for the viral genome to localize to cellular genomic DDR sites to jumpstart its replication. NS1 bound to heterologous DNA molecules containing NS1 consensus-binding elements are sufficient to transport DNA molecules to cellular DDR sites, suggesting a novel transport function for MVM-NS1. MVM initiates infection in the early S-phase, inducing additional cellular DNA damage as it amplifies. Using single-molecule DNA fiber assays, we discovered that cellular replication stress is induced prior to the initiation of virus replication. The presence of non-replicating MVM genome in the nucleus is sufficient to induce the shortening of host replication fibers, suggesting the viral genome perturbs cellular DDR pathways prior to the start of replication, implicating the single-stranded viral genome in the induction of cellular replication stress. 

### 5.6. Carnivore Bocaparvovirus 1 (Minute Virus of Canines) NP1 Modulates Viral Alternative RNA Processing

*Lisa Uhl, Yaming Dong, David J. Pintel *and* Olufemi Fasina*

Post-transcriptional mRNA regulation is a critical cellular homeostatic node often hijacked by viruses for a productive life cycle and, at present, is utilized for the design and development of severe acute respiratory coronavirus 2 (SARS-CoV-2) vaccines. Parvoviruses are linear single-stranded DNA viruses and use extensive RNA processing strategies to maximize their coding capacity. This provides an excellent tractable model to elucidate viral RNA processing mechanisms and host interactions geared toward the development of efficient parvovirus gene therapy and oncolytic virotherapy. Carnivore bocaparvovirus 1 (minute virus of canines, MVC) is an autonomous parvovirus in the Bocaparvovirus genus. It has a single promoter that generates a single pre-mRNA that is processed via alternative splicing and alternative polyadenylation to generate at least eight mRNA transcripts. MVC contains two polyadenylation sites, one at the right-hand end (pA)d and another (pA)p that lies within the capsid-coding region. Our previous results showed that the parvovirus non-structural protein NP1 modulates alternative splicing and alternative polyadenylation for efficient capsid production. The mass spectrometry analysis of NP1 interactome revealed a potential interaction with RNA-processing proteins, DNA damage response proteins and chromatin-remodeling proteins. We present the current data addressing the NP1 protein features and potential mechanisms utilized to modulate the alternative processing of MVC RNA. In addition, we address its potential role in nuclear domain reorganization, enabling the formation of nuclear compartments viral RNA processing and viral genome replication. NP1 is the first parvovirus protein implicated in RNA processing, and its discovery provides an excellent model to understand the dynamics and complex regulation of viral RNA processing and viral genome replication in an excellent tractable system.

### 5.7. A Functional Minigenome of Parvovirus B19

*Alessandro Reggiani, Erika Fasano, Gloria Bua, Elisabetta Manaresi *and* Giorgio Gallinella*

Parvovirus B19 (B19V) is a human pathogenic virus of clinical relevance, characterized by a selective tropism for erythroid progenitor cells in the bone marrow. The study of viral characteristics and lifecycle has always been limited by the availability of just two cellular systems able to support viral replication, the UT7/EpoS1 cell line and PBMC-derived EPCs, and by the difficulties in obtaining high-titer viral replication, hence the need to use patient-derived sera as the viral stock. Relevant information can thus be obtained from experiments involving engineered genetic systems in appropriate in vitro cellular models. Recently, a new model was developed in our laboratory: a genotype 1a consensus sequence was used to generate a genomic clone, named CK, which can replicate in vitro after the nucleofection of UT7/EpoS1 cells and lead to an infectious viral progeny that can be propagated by infecting EPCs, thus reducing the need of clinical samples and improve the manageability of B19V model systems.

In this project, with the dual aim of testing the plasticity of the B19V genome to rearrangements and focusing our attention on the NS1 protein, we design and produce a derived B19V minigenome reduced to a replicon unit. The genome terminal regions were maintained in a form able to sustain viral replication, while the internal region was modified by the deletion of the right-hand half of the internal region and the generation of a new cleavage-polyadenylation site, thus maintaining the coding sequence for the functional NS1 protein. Following transfection in UT7/EpoS1 cells, this minigenome still proved competent for the replication, transcription and production of NS1 protein. Furthermore, the B19V minigenome was able to complement B19-derived, NS1-defective genomes, restoring their ability to express viral capsid proteins. The B19V genome was thus engineered to yield a two-component system with complementing functions, providing a proof of concept of B19V genome-editing possibilities and, at the same time, a useful tool to study the NS1 protein also as an antiviral target.

## 6. Session 4: Trafficking

### 6.1. Review Lecture: Nuclear Entry and Egress of Parvoviruses

*Maija Vihinen-Ranta*

After the endosomal low-pH-induced exposure of the nuclear localization sequence on the capsid surface and escaping into the cytosol, parvovirus capsids are destined to enter the nucleus. Due to the small capsid diameter of 18–26 nm, intact capsids are imported actively to the nucleus through nuclear pore complexes (NPCs). Capsids may also use an alternative NPC-independent nuclear entry pathway that includes the activation of mitosis and the disruption of the nuclear envelope. Once the viral genome is released in the nucleus, viral replication compartments are initiated and the infection proceeds. The viral genome is replicated during the cellular S-phase followed by capsid assembly during virus-induced G2/M cell cycle arrest. The active nuclear egress of progeny capsids through the NPC is mediated by the chromosome region maintenance 1 protein, CRM1, and is enhanced by the phosphorylation of the N-terminal domain of VP2. Alternatively, capsid exit from the nucleus is facilitated by virus-induced transition to mitosis and apoptosis, which leads to an increase in NE permeability. 

### 6.2. A Classical cNLS Is Responsible for the Importin α/β-Dependent Nuclear Transport of Human Parvovirus B19 Non-Structural Protein 1

*Gualtiero Alvisi, Elisabetta Manaresi, Emily M. Cross, Nasim Akbari, Gayle F. Petersen, Roberto Garuti, Jade Forwood *and* Giorgio Gallinella*

Human parvovirus B19 (B19V) is a major human pathogen that causes a variety of diseases, ranging from fifth disease in children to pure red cell aplasia in immunocompromised individuals, and that is still in need of effective antiviral strategies. In similar fa ashion to all *Parvoviridae* members, the B19V small ssDNA genome is replicated within the nucleus of infected cells through a process that involves both cellular and viral proteins. Among the latter, a crucial role is played by the non-structural protein (NS)1, a multifunctional protein involved in genome replication and transcription, as well as the modulation of host gene expression. Despite the fact that NS1 can be visualized in the host cell nucleus during infection, its nuclear transport pathway and the nuclear localization signal (NLS) have not been characterized to date. In this study, we apply a combination of biochemical and imaging assays to demonstrate that NS1 is actively translocated in the cell nucleus by the importin (IMP) α/β heterodimer in an energy- and Ran-dependent process. Nuclear transport appears to be completely dependent on a short sequence of amino acids 172-GACHAKKPRIT-182, which binds IMPα with nanomolar affinity and confers active nuclear accumulation to GFP and, therefore, represents a classical (c)NLS. Interestingly, the K177T substitution completely abrogated IMPα binding and nuclear import. Furthermore, treatment with Ivermectin, an antiparasitic drug interfering with the IMP α/β-dependent nuclear import pathway, inhibited viral replication in infected UT7 cells. Thus, the NS1 nuclear transport emerges as a potential target of therapeutic intervention against B19V-induced disease.

### 6.3. Adeno-Associated Virus Type 2 (AAV2) Uncoating Is a Stepwise Process and Is Linked to the Structural Reorganization of the Nucleolus

*Sereina O. Sutter, Anouk Lkharrazi, Elisabeth M. Schraner, Anita F. Meier, Kevin Michaelson, Hildegard Büning *and* Cornel Fraefel*

Nucleoli are membrane-less nuclear structures formed by liquid–liquid phase separation and are known to be involved in many cellular functions, such as ribosomal RNA (rRNA) synthesis, ribosome biogenesis, stress response and cell cycle regulation. Many viruses can employ the nucleolus or nucleolar proteins to promote different steps of their life cycle, including replication, transcription and assembly. While adeno-associated virus type 2 (AAV2) capsids have previously been reported to enter the host cell nucleus and accumulate in the nucleolus, both the role of the nucleolus in AAV2 infection and the viral uncoating mechanism remain elusive. To elucidate the properties of the nucleolus during AAV2 infection and to assess viral uncoating at a single-cell level, we combined immunofluorescence analysis for the detection of intact AAV2 capsids and capsid proteins with fluorescence in situ hybridization for the detection of AAV2 genomes. The results of our experiments provide evidence that the uncoating of AAV2 particles occurs in a stepwise process that is completed in the nucleolus and supported by the alteration of the nucleolar structure.

### 6.4. The Phase-Separation Properties of the AAV2 Assembly-Activating Protein

*Janine Vetter, Manuel Kley, Catherine Eichwald *and* Cornel Fraefel*

Adeno-associated virus 2 (AAV2) is a helper-virus-dependent non-pathogenic Dependoparvovirus studied extensively due to its potential as a gene delivery vector. The 4.7 kb single-stranded DNA genome of AAV2 consists of two coding regions termed *rep* and *cap*, flanked by non-coding inverted terminal repeats. A +1 open reading frame within the *cap* gene encodes a non-structural protein of 204 amino acids named assembly-activating protein (AAP2), which has been attributed a critical role in transporting the viral capsid protein VP3 into the nucleolus for assembly. In the absence of other viral proteins, AAP2 localizes to the nucleolus due to five redundant nuclear and nucleolar localization signals. However, AAP2 remains poorly characterized because of its relatively late discovery and lack of commercial antibodies. Using AAP2 fusions to red or green fluorescent proteins, we assessed the liquid–liquid phase separation (LLPS) properties of AAP2 in transfected cells. Interestingly, AAP2 formed liquid-like condensates in a dose-dependent manner. Similar to other proteins that drive LLPS, fluorescence recovery after photobleaching (FRAP) revealed rapid turnover rates between AAP2-mKO condensates and the nuclear environment. Furthermore, we show that treatment with aliphatic diols, commonly used tools for the characterization of biomolecular condensates, led to the dissolution of AAP2 inclusions. Lastly, we observed AAP2 condensates in cells co-infected with AAV2 and HSV-1. Taken together, these data indicate that AAP2 confers liquid-like properties to viral compartments. However, the role of AAP2 LLPS properties in AAV2 replication remains to be elucidated.

## 7. Session 5: Virus–Host Interactions

### 7.1. Identification of Human Monoclonal Antibodies Potently Neutralizing Parvovirus B19

*Davide Corti*

Infection with Parvovirus B19 is associated with the common childhood disease erythema infectiosum, but also to more severe diseases, including hydrops fetalis, aplastic crises, polyarthralgia and pure red-cell aplasia. The current treatment for patients suffering from persistent B19 virus infection is the administration of high doses of human immunoglobulin preparations (IVIGs). We describe the isolation of a panel of 15 B19-specific monoclonal antibodies directed to multiple antigenic sites on VP1 and VP2. Two antibodies specific for VP1 and VP2 were selected for their neutralizing potency, which was found to be more than 1000-fold higher than IVIGs and mapped to two distinct sites on VP1 and VP2. A cocktail of these two antibodies may represent the basis to develop a novel therapeutic to treat severe B19 infections.

### 7.2. Enhanced Detection and Production of Parvovirus B19V Depends on the Cell Cycle Status of Erythroid Cells

*Zahra Kadri, Amandine Langelé, Bruno You, Céline Ducloux, Olivier Goupille, Emmanuel Payen *and* Stany Chrétien*

Human parvovirus B19 (B19V) causes diseases with severities ranging from benign childhood illness to arthropathies, severe anemia or hydrops fetalis, depending on the age, health and hematological status of the patients. Although the propagation of the virus is air-borne or vertical, blood-borne transmission remains possible, especially by the use of labile products. To assess the presence of B19V, the detection of B19V DNA is performed in plasma pools and at each step of the preparation of labile products. However, the detection of B19 nucleic acid does not predict B19V infectivity. Thus, at present, infectious titration systems using animal parvoviruses are used to evaluate the efficacy of inactivation/reduction steps during plasma processing, even though they imperfectly reflect human B19V infectivity. These methods have thus to be revised. Human cell lines permissive to B19V are available (e.g., UT7/Epo-S1), but substantial modifications are required to enable them to detect the presence of viruses and to produce large amounts of infectious viral particles.

We report the improvement of the detection of B19 infectious particles through the quantification of VP1/2 RNA expression and the evaluation of B19 infectious particles’ production in selected permissive cells. Of all our tested erythroid cell lines, our UT7/Epo cells (named UT7/Epo-STI) showed the highest sensitivity to B19 infection. We generated stable sub-clones of it and selected those with the highest permissiveness for B19V. Using the FUCCI (fluorescent ubiquitination cell cycle indicator) expression system, we show the direct correlation between infectivity and the cell cycle status of the cells. RNA sequencing showed that new cell lines were significantly different from the previously established UT7/Epo-S1 reference cell clone. The two sub-clones UT7/Epo-B12 and UT7/Epo-E2 had B19V detection and B19 infectious unit production capacities comparable to those obtained with CD36^+^ primary erythroid progenitor cells, the natural host cells for B19V, and 35-fold higher capacity than the UT7/Epo-S1 cell line. Indeed, 96 h after the inoculation of UT7/Epo-E2 cells by B19V, up to 10^9^ genome equivalent/mL of supernatant were obtained and the B19 infectious units’ production reached more than 10^4^ TCID_50_/mL, a level comparable to that obtained with CD36^+^ cells.

### 7.3. Rodent Protoparvoviruses MVMp and H-1PV Are Master Regulators of the Host Antiviral Innate Immune Response

*Assia Angelova, Annabel Grewenig, Estelle Santiago, Jürg Nüesch, Jean Rommelaere *and* Laurent Daeffler*

Oncolytic rodent protoparvoviruses (PVs) MVMp and H-1PV are considered attractive candidates for cancer therapy since, in addition to exhibiting oncolytic activity, they also drive some anticancer immune responses (AIRs). The production of type-I interferons (IFN) is considered instrumental for the activation of an efficient AIR by immunotherapies. Viruses can achieve IFN production in their host through the detection of viral factors (pathogen-associated molecular patterns, PAMPs) by the cellular receptors (pattern recognition receptors, PRRs) of the antiviral innate machinery. PVs were shown to induce IFN production in normal (mouse embryonic fibroblasts (MEFs) and human peripheral blood mononuclear cells (PBMCs)), but not in transformed/tumor cells, a feature that may therefore limit their immunostimulatory and oncosuppressive potential. The present study shows that the IFN production triggered by MVMp in MEFs requires some level of PV replication. We prove, moreover, that the PV infections of normal but also transformed/tumor cells activate the PRR pathway and that they also produce molecules that have the potential to behave as PRR ligands (PAMPs). Taken together, our data indicate that replicative PV infections are sensed by the innate immune machinery of their hosts, but that a mechanism is triggered by the virus, specifically in neoplastic cells, to prevent IFN production (evasion mechanism). In agreement with this hypothesis, we observed that the pre-infection of transformed/tumor but not normal cells with MVMp or H-1PV prevent a further IFN production triggered by classical PRR ligands, including Poly(I:C) and Newcastle disease virus (NDV). Altogether, we believe that our results will pave the way for the development of second-generation mutant PVs, devoid of this evasion mechanism and endowed, therefore, with an increased immunostimulatory potential through their ability to induce IFN production in infected malignant cells.

### 7.4. Parvovirus Infection Alters the Nucleolar Structure

*Salla Mattola, Simon Leclerc, Satu Hakanen, Vesa Aho, Colin R. Parrish *and* Maija Vihinen-Ranta*

The nucleolus is a dynamic nuclear structure that plays important roles in ribosome biogenesis and cellular stress response to stressors, such as viral infections. The nucleolus and nucleolar proteins are essential for the progression of infection by several viruses. Consequently, viral infection often induces alterations in the nucleolar structure and composition. We apply a deep learning algorithm segmentation and nucleolin labeling to analyze the nucleolar changes induced by autonomous parvovirus infection. Our results show that the size of nucleoli decreases and nucleolin is released into the nucleoplasm in late infection. The analysis of ki-67, one of the NS2-associated nucleolar proteins and a key factor in nucleolar organization, showed that the interaction between ki-67 and the DNA increases during infection. The infection initiated by a viral clone lacking an intact NS2 failed to decrease the nucleolar size; however, the orientation of the nucleoli changed. Our results suggest that parvoviruses modify and exploit nucleoli and nucleolar proteins during infection, and NS2 protein might play a role in the regulation of these processes

### 7.5. Parvovirus Non-Structural Protein 2 Associates with Chromatin-Regulating Proteins

*Salla Mattola, Kari Salokas, Vesa Aho, Elina Mäntylä, Sami Salminen, Satu Hakanen, Colin R. Parrish, Markku Varjosalo *and* Maija Vihinen-Ranta*

Autonomic parvoviruses encode only a limited number of proteins, making them highly dependent on the functions provided by the host cell. While the non-structural protein 1 is linked to critical nuclear processes required for viral replication, considerably less is known about the role of the non-structural protein 2 (NS2). Specifically, the function of canine parvovirus (CPV) NS2 remains undefined. We used mass spectrometry-based proximity-dependent biotin identification (BioID) to identify the proteins associated with nuclear CPV NS2. Most of the identified proteins were observed in both noninfected and infected cells. However, the location of the interacting proteins shifted from nuclear envelope proteins to chromatin-associated proteins in the infected cells. BioID high-confidence interactions revealed a potential role for NS2 in DNA remodeling and damage response. Further protein–protein interaction analysis by a proximity ligation assay confirmed the nuclear interactions of NS2 with selected key proteins identified by the BioID analysis. Mutations to the NS2 splice donor and acceptor sites affected chromatin organization and DDR-related proteins in infection. Additionally, the mutation of the NS2 splice donor site led to the deficient formation of replication centers. Our findings suggest that CPV NS2 has previously undiscovered roles in the interplay with cellular machinery regulating chromatin functions, replication center formation and DNA damage.

## 8. Session 6: AAV and Viral Vectors

### 8.1. Detection of AAV2 in Living Cells

*Luisa F. Bustamante-Jaramillo, Josh Fingall *and* Michael Kann*

AAVs are non-pathogenic members of the *Parvovirus* family used in gene therapy. They are mostly applied as self-complementary double-stranded vectors allowing to bypass the wild-type AAVs’ requirement of co-infection by a helper virus (herpes or adenovirus). Despite of their use as EMA- and FDA-accredited drugs, their application is hampered by their poor capacity to transduce cells and doses of 10^13^ particles per kg have to be administered. This limitation led us to search for factors restricting scAAV infection with the goal of improving gene therapy, focusing on the poorly understood steps of viral entry and viral genome release. In order to visualize the genomes released from the viral capsid, we used the so-called anchor system, which is based on the accumulation of >400 exogenously expressed fusion proteins, named OR-EGFP, on a double-stranded DNA seed sequence, allowing to detect single DNA sequences by time-lapse microscopy. EGFP-OR binding is dynamic, with a half-life of 52 s, allowing the expression of flanking genes. In our system, we inserted the anchor sequence flanked by m-Cherry ORF under the control of the P_CMV_ promotor into the AAV2 genome. As the dynamic binding of OR-EGFP to the anchor sequence allows the restoration of bleached fluorescent OR-EGFP, we followed the isolated capsid-released scAAV2 genomes and m-Cherry expression for 48 h in real time after the transduction of OR-EGFP-expressing cells. Using this approach, we obtained the kinetics of genome release and protein expression in single cells, also allowing investigations on the cellular factors involved in genome release by using inhibitors, such as e.g., Thapsigargin, that inhibit calcium accumulation in the ER and D-I03, thus blocking Rad52.

### 8.2. Whole-Genome siRNA and microRNA High-Throughput Screenings to Identify the Molecular Determinants That Govern AAV Vector Transduction

*Lorena Zentilin, Edoardo Schneider, Ambra Cappelletto *and* Mauro Giacca*

Adeno-associated viral vectors (AAVs) have emerged as leading gene delivery vectors to treat various inherited, degenerative or acquired conditions. However, despite the patent efficacy of AAV vectors in transducing several tissues in vivo and in vitro, incomplete information is available on the molecular determinants of AAV tissue permissivity. 

We previously observed that the host cell DNA damage response machinery and, in particular, the proteins involved in double-stranded DNA break repair (DSB) (including the Mre11–Rad50–Nbs1–MRN complex) interact and negatively regulate incoming AAV genome processing. To systematically identify the host cell factors involved in internalization, intracellular trafficking, the processing of AAV genomes and, eventually, AAV gene expression, either positively or negatively, we previously performed a high-throughput screening using a genome-wide siRNA library (over 18,000 genes were analyzed; Mano et al. PNAS 2015). We also performed a high-throughput screening using a genome-wide library of human microRNA mimics (988 mature sequences, miRBase 13—Dharmacon) in AAV2 Luciferase-transduced HeLa cells. Using this approach, we found that 51 microRNA mimics increased AAV transduction more than 4-fold (up to 23-fold change), while 26 microRNA mimics significantly decreased AAV efficiency. 

Hsa-miR-329 and hsa-miR-362-3p, which share the same seed sequence, were identified as the most effective microRNAs when increasing AAV transduction in HeLa cells. However, we also observed that the extent of the effects of the miRNAs on AAV transduction is dependent on the cell line tested, underlining the complexity of the vector–cell interaction. Transcriptomic analysis on the total RNA extracted from HeLa cells transfected with the three miRNAs identified several hundred transcripts that were significantly downregulated. The direct comparison of these results with those obtained from the whole-genome siRNA screening revealed the involvement of proteins that interfere with virus endocytosis or nuclear import or act in intrinsic cellular defense mechanisms. We are confident that a better understanding of the mechanisms of action of the identified genes and RNAs will open the possibility of developing antisense or RNAi-based RNA treatments to improve in vivo AAV vector transduction. 

### 8.3. From AAV Virus to AAV Vectors: Characterization of a Collection of AAV Capsid Variants Isolated from the Human Liver

*T. La Bella, B. Bertin, J. Nozi, T. Tedesco, A. Mihaljevic, P. Vidal, S. Imbeaud, N.C. Nault, J. Zucman-Rossi *and* G. Ronzitti*

Adeno-associated viral (AAV) vectors represent the leading platform for gene therapy in several genetic diseases. One of the key advantages of AAV vectors is their high versatility: thirteen AAV serotypes and hundreds of naturally occurring variants have been identified, each with varying transduction properties. In 2020, we screened a cohort of 1319 human non-tumor liver tissues to isolate 59 new capsid variants (La Bella T, Imbeaud S et al. 2020). Two distinct AAV subtypes were identified: one similar to AAV2 and the other a hybrid between the AAV2 and AAV13 sequences. The wild-type (wt) AAV2 variants belonged to clade B of AAV classification, whereas the wt AAV2-13 variants were closer to the sequences from clade C. The newly identified capsids were cloned into a plasmid suitable for AAV vector production and the vectors were characterized in terms of manufacturability, in vitro and in vivo transduction efficiency. A total of 70% of the capsids passed the first selection criteria based on manufacturing yields and was infectious in cells. In vivo biodistribution, assessed after intravenous injection in C57BL/6 mice, revealed a marked tropism for muscles for the majority of the capsids, opening the way for a possible muscle-directed AAV gene therapy application. Interestingly, some of the capsids were able to reach the central nervous system (CNS), suggesting a capacity to bypass the blood–brain barrier, a remarkable feature for CNS transduction via systemic administration. In order to understand the impact the amino acid variations on AAV capsid production and transduction efficiency, an analysis of mutations in structural and non-structural proteins was performed, leading to the identification of some indels within the VP sequences, which might be responsible of the destabilization of the VP structure. Moreover, mutations in AAP and protein X genes were identified, suggesting a possible impact on vector production yields.

### 8.4. Isolation of Novel AAV Serotypes from Pig Tissues for Retinal Gene Therapy

*Emanuela Pone, Vivien Temás, Antonella Ferrara, Ferenc Olasz, Anna Furiano, Ivana Trapani, Zadori Zoltán *and* Alberto Auricchio*

Due to their excellent efficacy and safety profile, vectors derived from the adeno-associated virus (AAV) are the leading platform for retinal gene therapy. The PCR-based isolation of AAVs from biological samples represents an important source of new AAV variants with unique biological features. In our study, we aim at isolating, from pig tissues, novel AAV variants with potential improved retinal transduction capabilities compared to the existing ones. To this aim, we screened by PCR porcine blood (*n* = 244) and liver (*n* = 274) tissues for the presence of AAVs, using primers complementary to conserved and flanking hypervariable regions in the *CAP* gene. We found that about 23% of blood and 28% of liver samples contained AAV sequences. Aminoacidic sequence alignment revealed five novel AAV variants from blood, which have 85–97% homology with the closest porcine AAV5 (AAVpo5) capsid sequence. One of them, AAVpoaa1, transduced pigs’ retinal pigmented epithelium and photoreceptors to levels comparable to the gold-standard AAV8 upon subretinal injection. The isolation, cloning and characterization of additional variants of pig origin is ongoing.

### 8.5. Development of a Synthetic AAV Vector for the Gene Therapy of Hemophilia in Children

*Jakob Shoti, Keyun Qing *and* Arun Srivastava*

In all previous or currently ongoing clinical trials for the gene therapy of hemophilia, children have not been enrolled since traditional liver-directed AAV gene therapy is unlikely to work for the following reasons: (i) Hepatocytes in the growing liver undergo rapid cell divisions, and with every cell division, the AAV vector genomes would be expected to be diluted due to the episomal nature of the AAV genome; and (ii) Repeat vector dosing is not an option since, following the first administration of the vectors, neutralizing antibodies are generated, making subsequent vector delivery difficult. We previously reported the development of a synthetic AAV vector, named No-End (NE) AAV DNA (*Gene*, 119: 265–272, 1992), which is devoid of AAV capsid proteins and which, upon transfection into human hepatic cells, leads to the sustained expression of a reporter EGFP gene for up to 35 days (*Mol Ther*, 29: 178, 2021). In the present studies, an optimized NE-DNA containing the human Factor IX (hF.IX) gene expression cassette, under the control of a human liver-specific transthyretin (TTR) promoter (optNE-TR75-TTR-hF.IX), was generated. This NE-DNA was transfected into a human hepatocellular carcinoma cell line, HepG2. The same expression cassette as that of a linear DNA was used as an appropriate control. Following transfections, the cells were passaged for up to 28 days. Cell extracts were prepared from replicate cultures and equivalent amounts of total cellular proteins were analyzed on Western blots using a hF.IX-specific monoclonal antibody. These results indicate that ~6-fold higher hF.IX levels were expressed from the optNE-TR75-TTR-hF.IX cassette compared with those from its linear counterpart up to 28 days post-transfection. These data are consistent with our previous studies with NE-DNA containing a reporter gene, suggesting that the observed increased hF.IX gene expression is due to the prolonged stability of the NE-DNA. Studies are currently underway to generate optNE-TTR- DNA containing the human clotting factor VIII (hF.VIII). In future studies, the encapsidation of these optNE-DNAs in liver-targeted synthetic liposomes may provide a viable approach for repeated delivery, and thus, a potential gene therapy of hemophilia in children. (This research was supported by a 2021 Global Hemophilia ASPIRE grant from Pfizer)

## 9. Session 7: Clinical and Veterinarian Parvovirology

### 9.1. First Evidence of a New Porcine Parvovirus Species in Italy: A Survey of Reproductive Failure Outbreaks

*G. Faustini, C.M. Tucciarone, A. Donneschi, G. Franzo, B. Boniotti, G.L. Alborali and M. Drigo*

An epidemiological update on the porcine parvovirus 1 (PPV1) presence and the first evidence of a newly identified species (PPV2-7) circulation in Italy is presented in this paper, filling the current lack of knowledge. Fetuses collected from reproductive failure outbreaks in Northern Italian farms and submitted to the Istituto Zooprofilattico Sperimentale della Lombardia e dell’Emilia Romagna (IZSLER) in the period of 2019–2020 were tested by using four multiplex real-time PCRs, described in previous studies.

PPV1 and new PPV species were respectively detected in 23.8% and 42.5% of the tested farms (*n* = 80), respectively. Considering other pathogens routinely investigated by IZSLER, PPV2-7 co-infections with viral and bacterial agents were observed in 25.0% and 12.5% of the farms, respectively. PCV-2 and at least one PPV2-7 species were the most common co-infections (17.5%). PPV1 and new PPVs were detected in 5% and 15% of the farms, respectively. PPV detection singularly or in co-infection with other pathogens commonly responsible for reproductive failure encourages future studies investigating their biological, clinical and epidemiological roles, for a better preparedness to potential future challenges.

### 9.2. Phylogenetic and Clinical Relationships among Canine and Feline Parvovirus Strains Collected from Parvovirosis Cases in Italy

*Claudia Maria Tucciarone, Giovanni Franzo, Matteo Legnardi, Andrea Zoia, Matteo Petini, Tommaso Furlanello, Marco Caldin, Mattia Cecchinato *and* Michele Drigo*

*Carnivore protoparvovirus 1* is a relevant species in veterinary medicine that includes canine (CPV) and feline parvovirus (FPV), which can cause severe gastroenteritis and immunosuppression, especially in young animals. CPV heterogeneity has been extensively studied since the discovery of its antigenic variants, to which different virulence levels are occasionally attributed. On the other hand, FPV has been considered less variable and less rapidly evolving than CPV.

Based on these premises, full VP2 sequences were obtained from Italian clinical cases of parvovirosis in dogs and cats. CPV and FPV molecular epidemiology was described by phylogenetic analyses, highlighting the viral heterogeneity and variant exchange within and among countries. Moreover, the relationship between the CPV and FPV strains with the clinicopathological outcome was statistically investigated, revealing that viral phylogeny is associated with host parameters of inflammatory response, even though a statistical significance was proven only for CPV, whose higher heterogeneity likely determined a stronger phylogenetic signal. These results suggest a more complex contribution of viral phylogeny rather than of antigenic features in determining severity.

### 9.3. Post-Vaccination Polyclonal Antibody Response Mapped by Cryo-EM

*Samantha R. Hartmann, Simon Frueh, Robert Lopez-Astacio, Wendy S. Weichart, Nadia Di Nunno, Sung Hung Cho, Carol M. Bato, Colin R. Parrish *and* Susan L. Hafenstein*

Canine parvovirus (CPV) is an important pathogen that emerged by cross-species transmission and causes severe disease in dogs, including acute hemorrhagic enteritis, myocarditis and cerebellar disorder. There is overlap on the surface of parvovirus capsids between the A- and B-site antigenic epitopes and the binding site for the host receptor, the transferrin receptor-1 (TfR). Selection pressure by antibodies or TfR has led to closely related variants that differ in antigenicity and host range. Nevertheless, vaccination to canine parvovirus is very effective. To understand the host immune response to vaccination, serum from dogs immunized with live parvovirus were obtained; the polyclonal antibodies were purified and used to obtain the high-resolution cryo-EM structures of the polyclonal Fab-virus complexes. We used a custom software, Icosahedral Subparticle Extraction and Correlated Classification (ISECC), to perform sub-particle analysis and reconstruct polyclonal Fab-virus complexes from two different dogs eight and twelve weeks post-vaccination. In the resulting polyclonal Fab-virus complexes, there were a total of five distinct Fabs identified, directed to the A- and B-sites. Both dogs generated an antibody that recognized the B epitope with identical footprints onto the virus capsid. In both cases, all five antibodies identified would interfere with receptor binding. This polyclonal mapping approach identifies a specific, limited immune response to the live vaccine virus and provides a new method for using cryo-EM to investigate the complex binding of multiple different antibodies or ligands to virus capsids.

### 9.4. Human Parvovirus Tissue Persistence: Cell Tropism, Activity and Impact

*Man Xu, Katarzyna Leskinen, Tommaso Gritti, Valerija Groma, Johanna Arola, Anna Lepistö, Taina Sipponen, Päivi Saavalainen *and* Maria Söderlund-Venermo*

Two parvoviruses have been discovered as human pathogens to date, parvovirus B19 and human bocavirus (HBoV) 1. B19V shows extreme tropism to erythroid progenitor cells, resulting in erythema infectiosum, anemia and fetal death. HBoV1 replicates in human airway epithelial cells and causes respiratory tract infections, while HBoV2-4 is enteric. After primary infection, B19V DNA persists in a wide variety of tissues lifelong, whereas HBoVs persist in some. We aim to determine the host cell tropism, virus activity and impact of human parvoviruses in non-permissive host cells. In pediatric tonsillar tissues, we earlier discovered the persistent site of HBoV1 DNA to be lymphoid germinal centers (GCs) and the host cells to be naive, activated and memory B cells and monocytes. In both immortalized and primary tonsillar B-cell cultures, the cellular uptake of HBoV1 occurred through antibody-dependent enhancement (ADE) via the Fc receptor II. HBoV1 did not replicate productively in the tonsils nor in the ADE-mediated cell cultures, despite the detection of spliced mRNAs.

We searched for parvoviral DNA in biopsy specimens of paired diseased and healthy intestinal mucosa of 130 individuals, and detected persistent HBoV1-3 DNA in the intestines of only one individual in each case. Conversely, B19V DNA was detected in the cancerous, inflamed, adenomatous and healthy intestinal biopsy specimens of 50, 47, 31 and 27% of the individuals, respectively. Intra-individually, however, B19V DNA persisted significantly more often in the healthy than in the inflamed paired intestinal segments. By RNAscope-ISH and immunohistochemistry, we located the B19V DNA in the mucosal B cells of lymphoid follicles and vascular endothelial cells. The B19V transcription activity was, however, below the RT-PCR detection level. With RNA-seq analysis, we further identified 272 B19V-modulated, differentially expressed genes in normal ileum specimens, and B19V persistence was shown both to activate cell viability and to inhibit apoptosis. Life-long B19V DNA persistence thus seems to modulate host gene expression, which may lead to clinical outcomes in predisposed individuals.

### 9.5. High Prevalence and Activity of Cutavirus in Parapsoriasis Patients

*Ushanandini Mohanraj, Alexander Salava, Liisa Väkevä, Annamari Ranki *and* Maria Söderlund-Venermo*

In 2016, metagenomic studies in the diarrheic stool and skin of cutaneous T-cell lymphoma (CTCL) patients revealed a novel human protoparvovirus, cutavirus (CuV). Soon after, we and others detected CuV DNA in CTCL lesions, while all skin biopsy specimens of healthy subjects were CuV-DNA-negative, revealing an association of CuV with CTCL. Most patients with CTCL first present with long-standing reactive inflammatory conditions, such as parapsoriasis en plaques (a form of pre-CTCL). This has led to the hypothesis that chronic antigen stimulation by a pathogen could have a role in CTCL carcinogenesis. Hence, in the present study, we aim to identify the prevalence, activity and cell tropism of CuV in parapsoriasis skin lesions. 

We first studied 13 fresh skin biopsy specimens obtained from 12 patients (group A, age: 34–83 years). Of these 12 patients, 7 had CTCL and 5 had parapsoriasis. Next, we studied 24 FFPE (formalin-fixed, paraffin-embedded) skin biopsy samples and 52 skin swabs from 13 parapsoriasis patients (group B, age: 37–86 years). CuV was detected and quantified by qPCR and RT-qPCR. All DNA/mRNA-positive samples were confirmed by cloning and sequencing. By RNAscope in situ hybridization (RISH) and multiplex immunohistochemistry (mIHC), CuV DNA/RNA was visualized in the tissues of CuV DNA-positive individuals. In group A, CuV DNA and spliced mRNA were detected in freshly frozen skin biopsy specimens from 4/12 (33.3%) individuals. Among these four CuV-positive individuals, three had parapsoriasis (3/5, 60%) and one CTCL (1/7, 14.2%). In group B, CuV DNA was detected in swabs from 6/13 (46%) and in FFPE samples from 8/12 (66%) parapsoriasis individuals. Overall, among the 11 CuV-positive parapsoriasis individuals from both groups, 3 progressed to CTCL, while 1 had early stage CTCL. From the ISH + mIHC assays, CuV-specific DNA/RNA signals were observed in both the cytoplasm and nucleus of many cell types, including keratinocytes, T cells and macrophages. We found a very high CuV prevalence in the skin lesions of parapsoriasis en plaques. CuV also actively transcribed mRNA. Hence, it is imperative to clarify if skin-persistent CuV leads to T-cell stimulation and the development of CTCL and if CuV presence could serve as a prognostic marker for CTCL. 

## 10. Session 8: Gene Therapy

### 10.1. Dynorphin-Based “Release on Demand” Gene Therapy for Drug-Resistant Temporal Lobe Epilepsy

*Regine Heilbronn, Alexandra S. Agostinho, Mario Mietzsch, Luca Zangrandi, Iwona Kmiec, Anna Mutti, Larissa Kraus, Pawel Fidzinski, Ulf C. Schneider, Martin Holtkamp *and* Christoph Schwarzer*

Epilepsy represents one of the most common chronic CNS diseases, with temporal lobe epilepsy as the most frequent clinical presentation. The focus lies in the hippocampus, the site of learning memory and emotional control. The high incidence of drug resistance, devastating comorbidities and insufficient responsiveness to invasive epilepsy surgery are unmet medical challenges. In the quest for novel, disease-modifying treatment strategies, neuropeptides represent promising candidates. We recently provided “proof of concept” that the AAV-vector-based transduction of dynorphin into the epileptogenic focus of clinically well-accepted mouse and rat models for temporal lobe epilepsy leads to the suppression of seizures over months (EMBO Mol Med 2019, e9963). Also, the debilitating long-term decline in learning and memory is prevented. In human hippocampal slice cultures obtained from epilepsy surgery, dynorphins suppressed seizure-like activity, suggestive of their high potential for clinical translation. AAV-delivered neuronal dynorphin expression is focally restricted and its release is dependent on high-frequency stimulation, as it occurs at the onset of seizures. The novel format of the “release on demand” delivery of dynorphin is viewed as a key to prevent habituation and to minimize the risk of adverse effects, leading to the long-term suppression of seizures and of their devastating sequels. Progress on vector validation in the preparation of a clinical trial is presented.

### 10.2. Structural and Kinetic Characterization of Anti-AAV9 Monoclonal Antibodies Derived from Patients’ Post-Zolgensma^®^ Treatment

*Jane Hsi, Mario Mietzsch, Austin Nelson, Paul Chipman, Jenny Jackson, Peter Schofield, Daniel Christ, Joanne Reed, Neeta Khandekar, Grant Logan, Ian E. Alexander *and* Robert McKenna*

The use of adeno-associated virus (AAV) as a gene transfer vehicle has become increasingly feasible as a clinical option in recent years. Following the FDA approval of Zolgensma^®^ (Novartis), an AAV9-based biologic to treat children under the age of two with spinal muscular atrophy (SMA), there is a growing interest in expanding AAV9 usage due to its ability to transduce cardiac and skeletal muscle, liver, pancreas and eye and its capability to cross the blood–brain barrier to transduce the central nervous system (CNS). However, the presence of pre-existing neutralizing antibodies (NAbs) against AAV9 capsids in a large percentage of the population could reduce the efficacy of AAV9 gene therapy and may lead to the exclusion of patients from treatment. A strategy to circumvent the immune response of a patient for AAV-mediated therapeutic gene delivery is the development of engineered vector capsids by either directed evolution or rational design that are then able to escape antibody recognition. In order to pursue this strategy, the interactions of the NAbs to the capsid binding need to be characterized. Previously, our lab as well as others generated mouse monoclonal antibodies targeting the AAV9 capsids to simulate the immune response against the capsid and map the major antigenic regions. However, this approach has faced criticism as mouse-derived antibodies may not fully mimic the behavior of human-derived antibodies.

We present the structural and kinetic characterization of human monoclonal antibodies that were obtained from patients that received Zolgensma^®^. Specifically, we determine the binding sites of 13 antibodies obtained from two patients to the AAV9 capsid by cryo-electron microscopy and three-dimensional image reconstruction. Our data show that the 2-fold capsid surface is the antigenically dominant region as ~ three quarters of the antibodies bind there. The binding interactions and kinetics for these antibodies were analyzed using biolayer interferometry and compared to the previously developed murine antibodies. All the antibodies neutralize AAV9 transduction, and some also cross-react and neutralize a range of different AAV serotypes. Structural and kinetic comparisons of human and murine antibodies is presented along with implications for the development of antibody escape variants.

### 10.3. Development of Optimized (Opt) AAVrh74 Vectors with Increased Transduction Efficiency in Primary Human Skeletal Muscle Cells In Vitro and in Mouse Muscles In Vivo Following Systemic Administration

*Keyun Qing, Jakob Shoti, Geoffrey D. Keeler, Barry J. Byrne *and* Arun Srivastava*

In a Phase I/II clinical trial sponsored by Solid Biosciences using AAV9 vectors, serious adverse events were reported. In a trial sponsored by Pfizer, also using AAV9 vectors, several serious adverse events and the death of a patient were also reported. Sarepta Therapeutics reported the results of a Phase I/II trial using AAVrh74 vectors with vomiting as the only adverse event, indicating that AAVrh74 vectors are safer, although a high dose of 2 × 10^14^ vgs/kg was used. We previously reported that capsid-modified next-generation (“NextGen”) AAVrh74 vectors (*Mol. Ther.*, 29: 159–160, 2021) and genome-modified generation X (“GenX”) AAVrh74 vectors (*Mol. Ther.*, 29: 184–185, 2021) are significantly more efficient than their wild-type (WT) counterpart. In the present study, we combine the two modifications to generate optimized (“Opt”) AAVrh74 vectors and evaluate their transduction efficiency in primary human skeletal muscle cells in vitro, which was up to ~5-fold higher than that of the conventional AAVrh74 vectors. The efficacy of the WT and Opt AAVrh74 vectors was also evaluated in mouse muscles in vivo following systemic administration. The transduction efficiency of the Opt AAVrh74 vectors was ~5-fold higher in gastrocnemius (GA) as well as in tibialis anterior (TA) muscles. The total genome copy numbers of either the WT or Opt AAVrh74 vectors in GA, TA, diaphragm and heart muscles were not significantly different, suggesting that the observed increased transduction efficiency of the Opt AAVrh74 vectors was due to improved intracellular trafficking and nuclear transport of these vectors, which is consistent with our previously published studies with other Opt AAV serotype vectors. Taken together, these studies suggest that the use of Opt AAVrh74 vectors may lead to a safe and effective gene therapy of human muscular dystrophies at reduced doses. However, as observed in a recently published study (*Hum. Gene Ther.*, 32: 375–389, 2021), a significant fraction of the AAVrh74 vectors is sequestered in the liver, which is undesirable. Efforts are currently underway to develop liver de-targeted Opt AAVrh74 vectors for their optimal use in the gene therapy of human muscular dystrophies at further reduced doses. (This research was supported by a sponsored research grant from Sarepta Therapeutics).

### 10.4. Development of Genome-Modified Generation Y (GenY) AAVrh74 Vectors with Improved Transgene Expression in Primary Human Skeletal Muscle Cells In Vitro and in Mouse Muscles In Vivo Following Systemic Administration

*Jakob Shoti, Keyun Qing, Geoffrey D. Keeler, Barry J. Byrne *and* Arun Srivastava*

Since the naturally occurring AAV contains a single-stranded DNA genome and expresses viral genes poorly because ssDNA is transcriptionally inactive, transgene expression levels from recombinant ssAAV vectors are also negatively impacted. We previously reported that the substitution of the D-sequence in the left inverted terminal repeat (ITR) results in generation X (“GenX”) AAV vectors, which mediate up to 8-fold improved transgene expression (*J. Virol*., 89: 952–961, 2015). More recently, we also reported that the extent of transgene expression from GenX AAVrh74 vectors is ~5-fold higher than that from wild-type (WT) AAVrh74 vectors (*Mol. Ther.*, 29: 184–185, 2021). We previously observed that the distal 10-nucleotides (nts) in the AAV2 D-sequence share a partial homology to the consensus half-site of the glucocorticoid receptor-binding element (GRE), and that the glucocorticoid receptor signaling pathway is activated following AAV2 infection/AAV2 vector transduction (*Mol. Ther*., 24: S6, 2016). We evaluate whether the substitution of the distal 10 nts in the D-sequence with the authentic GRE leads to increased transgene expression from AAVrh74 vectors, named generation Y (“GenY”) vectors. The extent of the transgene expression from GenX and GenY AAVrh74 vectors in primary human skeletal muscle cells was, on average, respectively, ~4-fold and ~6-fold higher compared to that from the WT AAVrh74 vectors. The observed increase in transgene expression was not due to the increased entry of the GenX or the GenY vectors, as documented by approximately similar numbers of vector genome copy numbers quantitated by qPCR analyses of low mol. wt. DNA samples isolated from cells transduced with each of these vectors. The efficacy of the GenY AAVrh74 vectors in skeletal muscles following systemic administration in a murine model was also evaluated in vivo and shown to be higher than that from NextGen AAVrh74 vectors. These studies suggest that the combined use of the capsid-modified NextGen + GenY (Opt^Y^) AAVrh74 vectors may further reduce the need for the high vector doses currently in use, which has significant implications in the potential use of Opt^Y^ AAVrh74 vectors in the safe and effective gene therapy of muscular dystrophies. (This research was supported by a sponsored research grant from Sarepta Therapeutics)

## 11. Session 9: Oncolytic Viruses

### 11.1. Review Lecture: Characterization of the H-1PV Life Cycle as a Way to Improve Its Anticancer Efficacy

*Amit Kulkarni, Tiago Ferreira, Tiina Marttila, Gayatri Kawishwar, Anna Hartley *and* Antonio Marchini*

H-1 parvovirus (H-1PV) is the first member of the Parvoviridae family to be tested as an anticancer agent in early phases clinical studies. The results from these studies in patients with glioblastoma and pancreatic carcinomas showed that H-1PV treatment is safe, well-tolerated and associated with surrogate signs of efficacy. However, the virus alone, in the regimes applied, was unable to eradicate the tumors. The rational design of combination strategies, the development of more effective viruses and the identification of biomarkers that can predict which tumors are most likely to benefit from H-1PV-based therapies are ways to improve H-1PV anticancer efficacy. We review the strategies to improve H-1PV anticancer efficacy, with a special focus on the characterization of the pathways used by H-1PV to infect cancer cells and the identification of the cellular factors involved in virus cell attachment and entry. We propose that the expression levels of these critical modulators may guide the selection of patients whose tumors are more susceptible to H-1PV treatment.

### 11.2. Engineering Functional Domains of MVM Capsid with VEGF-Blocking Peptides to Enhance Parvovirus Oncolytic Capacity

*Tania Calvo-López, Esther Grueso, Cristina Sánchez-Martínez *and* José M. Almendral*

We attempt to direct the capsid of the prototype strain of the parvovirus minute virus of mice (MVMp) against the tumor vasculature using two strategies: (A) as a platform to induce α-VEGF antibodies and (B) by re-targeting the infection to cells expressing VEGF-R1. For this, peptides from the three functional domains of the MVMp capsid were replaced by peptides that block the binding of VEGF to its receptors (VEbp). (A) In the first approach, although most substitutions caused alterations in the assembly or maturation of the virus, some virus chimeras with VEbp substituting the residues at the 3-fold axis were efficiently propagated in the culture, exposed heterologous peptides on the surface of the capsid and induced native anti-VEGF antibodies in mice. (B) In the re-targeting studies, some virus chimeras bearing VEbp in the 2-fold axis, where the sialic-acid-receptor-binding site localizes, showed greater infectivity than MVMp towards human glioblastoma cells, although they did not infect through VEGF-R1. Instead, the chimeras attached, as MVMp, to distinct types of sialic acid (sia) residues. Our MVM re-targeting study illustrates: (i) the structural constrains to re-target parvoviruses that have evolutionary adopted narrow grooves to allocate small sia receptors; (ii) the critical role that attachment to specific types of sia plays in determining parvovirus; and (iii) the possibility to intervene the tropism of oncolytic parvoviruses by modulating the sia framework on the cancer cell surface.

### 11.3. MVM Selectively Infects and Disrupts Glioblastoma-Stem-Cell-Derived Tumors with Patient-Specific p53 Deregulations

*Jon Gil-Ranedo, Carlos Gallego-García *and* José M. Almendral*

Glioblastoma multiforme (GBM) remains a major type of cancer with no current effective therapy. Multiple studies support that the efficacy of cancer therapies must be primarily shown against the cancer stem cells driving tumorigenesis. We therefore attempt to develop a GBM therapy using two strains of the minute virus of mice (MVMp and MVMi), a parvovirus that is non-pathogenic for humans. Primary glioblastoma stem cells (GSCs) isolated from GBM patients were challenged with the MVM strains and the system comprehensively studied for cell and virus parameters. GSC neurospheres accumulated assembled capsids, but restrict viral NS1 cytotoxic protein expression by an innate PKR/eIF2α-P response. Inter- and intra-patient GSC heterogeneity was dissected by their diverse innate responses and the ratio between structural and non-structural viral gene expression. Viral infection triggered a comprehensive DNA damage response involving cell cycle arrest, neurosphere disorganization and the bystander disruption of GSC-derived brain tumor architecture in rodent models. Notably, the MVM infection preferably targeted those GSC subpopulations within patients harboring p53 gain-of-function mutants and/or Pp53Ser15 phosphorylation. This study supports MVM as an effective oncolytic agent for personalized parvoviral therapies against devastating glioblastoma and other cancers with deregulated p53 signaling.

### 11.4. Identification of an Antiviral Drug as a Novel Potentiator of H-1PV-Mediated Oncolysis

*Anna Hartley, Valérie Palissot, Tiina Marttila, Toros Tasgin, Céline Jeanty, Gian Mario Dore, Laurent Brino, Anne Maglott-Roth, Richard Harbottle *and* Antonio Marchini*

The rat protoparvovirus H-1 (H-1PV) is a small non-enveloped virus with a natural oncolytic capacity. Replication of the virus in human cells is strictly dependent on a transformed phenotype in the host cell, including a dependence on cellular S-phase factors. This has made H-1PV an attractive treatment option for human cancers. The oncolytic activity of H-1PV has been extensively tested in vitro against a wide range of cancer cell lines, including brain cancer, lymphoma and leukemia, pancreatic ductal adenocarcinoma (PDAC) and breast cancer. In the clinics, H-1PV as a monotherapy was shown to be safe and well-tolerated for the treatment of glioblastoma and PDAC, but it was unable to eradicate tumors under the regimes used. Thus, there is a clear need for the improvement of oncolytic H-1PV therapy. We identify a novel combination of H-1PV with an antiviral drug that potentiates the oncolytic capacity of the virus in vitro. 

We conducted a high-throughput screening of 1443 FDA-approved drugs in combination with H-1PV in glioma cells to identify drug candidates that are able to potentiate the oncolytic abilities of H-1PV. From this screening, we identified the hepatitis C virus inhibitor ledipasvir as a potentiator of H-1PV-mediated oncolysis and confirmed this in vitro in a variety of cellular backgrounds, including glioblastoma, PDAC, lung cancer and ovarian carcinoma. Importantly, this improved oncolysis was independent of viral replication. The functional kinome profiling (PamGene) in glioma cells co-treated with ledipasvir and H-1PV revealed a number of kinase pathways that are differentially activated in the presence of ledipasvir and H-1PV co-treatment. The causal role of these kinase pathways is under investigation at present. Our data demonstrate, for the first time, that ledipasvir is able to potentiate the oncotoxicity of H-1PV and is a promising candidate for combination therapy in the clinics.

### 11.5. Oncolytic H-1 Parvovirus in Combination with the Pro-Apoptotic Drug ABT-737 Shows Improved Cytotoxicity and Immune Activation in Prostate Cancer Cells

*Gayatri Kavishwar, Alice De Roia, Dirk M. Nettelbeck, Richard Harbottle, Marcelo Ehrlich, Guy Ungerechts *and* Antonio Marchini*

In the past decade, novel methodologies have been developed, facilitating the use of immune activation against cancer. This has greatly improved the overall survival and prognosis of various malignancies. However, not all cancer patients benefit from these treatments; therefore, there is an unmet need to find agents that could potentiate the efficacy of immunotherapy. One such field of research is oncolytic-virus-mediated immunotherapy. More than 40 oncolytic viruses (OVs) are presently undergoing clinical testing alone or, increasingly, in combination with other immunotherapeutic agents. OVs specifically target cancer cells, induce tumor cell lysis, tumor antigen release and adjuvant immune response through immunogenic cell death and cytokine release. H-1 parvovirus (H-1PV) is one such “clinically relevant” OV, which has been extensively studied in various cancer models and evaluated in Phase I/II clinical trials for the treatment of patients with glioblastoma and pancreatic ductal adenocarcinoma. It has been shown to be safe, non-toxic and capable of inducing the favorable immune modulation of the tumor microenvironment. However, H-1PV, as a monotherapy, is still not sufficient to completely eradicate the tumors. The development of combination strategies based on H-1PV and other anticancer agents thus seems to be a rational approach to improve efficacy. Previous data from our laboratory showed great promise in using the combination of H-1PV with pro-apoptotic BCL2 inhibitors in different cancer models. In this study, we evaluate whether it is possible to enhance the oncolytic activity of H-1PV by using the pro-apoptotic BH3 mimetic ABT-737 in prostate cancer cells. Our data show that this combination shows an improved killing of prostate cancer cells in a synergistic manner. We also provide first evidence that this cell death is immunogenic, by showing a strong induction of cell surface calreticulin, a classical marker of immunogenic cell death (ICD). To investigate whether the induction of an ICD event also causes the activation of the dendritic cell (DC)/T-cell axis, we established multiple co-culture systems using the prostate cancer cell line PC3 and primary immune cells. Firstly, we observed the maturation and activation of DCs through the upregulation of CD80 and CD86 expression. Furthermore, we observed the peripheral activation of T cells through the upregulation of the early activation marker CD69. The combination also shows an improved activation of natural killer cells through an increase in the degranulation marker CD107a and intracellular IFN𝛄. Our study thus shows that the combination treatment of H-1PV and ABT-737 shows promise in enhancing direct tumor cell killing as well as anticancer immunity in the context of prostate cancer.

## 12. Poster Session

A list of the submitted posters is reported below, while the full abstracts are available as Supplemental File S1.
**P.01 Variability analysis of Parvovirus B19 sequences obtained through Next-Generation Sequencing***Federica Bichicchi, Niccolò Guglietta, Arthur Daniel Rocha Alves, Gloria Bua, Francesca Bonvicini, Elisabetta Manaresi, Giorgio Gallinella***P.02 In vitro models for the study of B19V interaction with the human placental BeWo monolayer***Francesca Bonvicini, Gloria Bua, Erika Fasano, Elisabetta Manaresi, Giorgio Gallinella***P.03 Mesenchymal stem cells are susceptible but non-permissive to B19V replication***Gloria Bua, Pasquale Marrazzo, Francesco Alviano, Laura Bonsi, Giorgio Gallinella***P.04 The role of Host Cell Factor 1 in the life cycle of AAV***Caroline Dierckx, Zander Claes, Mathieu Bollen, Els Henckaerts***P.05 Analysis of Parvovirus B19 transcriptome in UT7/EpoS1 cells by mRNAseq techniques***Erika Fasano, Gloria Bua, Stefano Amadesi, Alessandro Reggiani, Elisabetta Manaresi, Fabrizio Ferrè, Giorgio Gallinella***P.06 Prevalence of Human Chaphamaparvoviruses***Jingjing Li, and Maria Söderlund-Venermo***P.07 Brain organoids as a platform to study subcellular trafficking of recombinant AAV vectors***Marlies Leysen, Idris Salmon, Sereina O. Sutter, Cornel Fraefel, Adrian Ranga, Benjamien Moeyaert, Els Henckaerts***P.08 Equine Parvovirus Hepatitis qPCR screening of stored equine heparin plasma and serum samples with and without increased liver enzyme activities***Anna Sophie Ramsauer, Ina Mersich, Irina Preining, Jessika-Maximiliane Cavalleri***P.09 FLIM-FRET studies of AAV capsid nuclear disintegration***Visa Ruokolainen, Michael Kann, Maija Vihinen-Ranta***P.10 Enhanced transduction efficiency of AAV vectors mediated by polyvinyl alcohol and human serum albumin is serotype- and cell type-specific***Jakob Shoti, Claire K. Scozzari, Reema Kashif, Hua Yang, Mengqun Tan, Wei Wang, Keyun Qing, Arun Srivastava***P.11 Development of allele-specific dual PCRs to identify members of the 27a cluster of PPV1***Vivien Tamás, Ferenc Olasz, István Mészáros, István Kiss, Zalán G. Homonnay, Preben Mortensen, Zoltán Zádori***P.12 A record of Parvovirus B19 laboratory diagnosis in endemic/epidemic or COVID-19 pandemic***Simona Venturoli; Alessia Bertoldi; Elisabetta Manaresi, Giorgio Gallinella.***P.13 Enhanced muscle transduction by clade F vector AAVhu.32 translates across multiple animal models***Samantha A. Yost, Randolph Qian, April R. Giles, Sungyeon Cho, Chunping Qiao, Olivier Danos, Ye Liu, Andrew C. Mercer*

## 13. Conclusions

The four days of sessions engaged attendees with an intense, but highly rewarding flow of information on the up-to-date and cutting-edge research in the field. Most of the abstracts presented already found their ways into the published literature, promoting research in the ever-fascinating field of Parvoviruses.

The two awards offered by the Journal *Viruses* were conferred to Jan Bieri (presentation 2.3) and Marlies Leysen (poster P.07).

**Figure 1:**
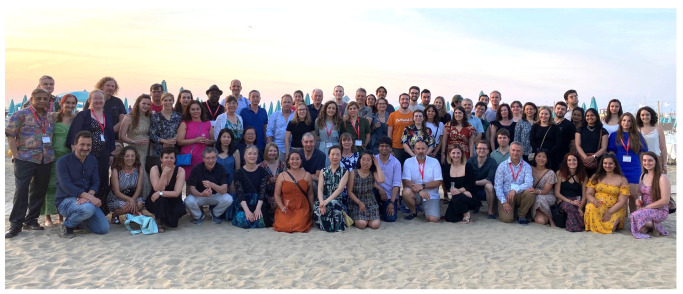
Rimini, 17 June 2022: relax on the beach (courtesy of Maria Söderlund-Venermo).

The closing event, a very informal dinner directly on the beach in Rimini (Figure 1), followed by wild dancing until dawn as in the best tradition, provided the link to the next XIX Parvovirus Workshop, which is to be held in Leuven, Belgium, from 3 to 6 September 2024, hosted by Els Henckaerts. Please visit: parvovirusworkshop2024.org.

## Data Availability

As a conference report, no new data were created or analyzed in this study. Data sharing is not applicable to this article.

## Supplementary Materials

The following supporting information can be downloaded at: https://www.mdpi.com/article/10.3390/v15102129/s1, Supplemental File S1: Poster Session.

