# Supplementary material for: The XVIII International Parvovirus Workshop Rimini, Italy, 14–17 June 2022
Source: Viruses. 2023 Oct 20;15(10):2129. doi: 10.3390/v15102129 (PMC10612026; doi:10.3390/v15102129)
Supplement: Supplementary file 1 [file viruses-15-02129-s001.zip › viruses-2691847-supplementary.pdf]

THE XVIII INTERNATIONAL PARVOVIRUS WORKSHOP

POSTER SESSION

P.01 Variability analysis of Parvovirus B19 sequences obtained through Next Generation Sequencing

Federica Bichicchi[1], Niccolò Guglietta[1], Arthur Daniel Rocha Alves[2], Gloria Bua[1], Francesca Bonvicini[1], Elisabetta Manaresi[1], Giorgio Gallinella[1]

[1] University of Bologna – Dept of Pharmacy and Biotechnology; [2] Oswaldo Cruz Foundation – Oswaldo Cruz Institute – Lab. of Technological Development in Virology, Rio de Janeiro, RJ, Brazil

Parvovirus B19 is a human pathogenic virus responsible of a wide range of clinical manifestations. Sequencing of viral genomes is frequently carried out for molecular epidemiological studies and possible correlation to diverse clinical manifestations. Through a Next Generation Sequencing approach, we aimed to investigate the presence of viral quasispecies in clinical samples. In quasispecies we observe a phenomenon in which low-frequency mutations form a mutant cloud of different viral genomes, and Next Generation Sequencing is required to understand the depth of variation in a quasispecies, which would be lost otherwise.

A total of thirty-four samples, identified as B19V positive in the course of institutional diagnostic service, have been selected for analysis. From these, amplification of the genomic segment between nt. 2210-3342 obtained by PCR. Amplification products were sequenced in paired-end 150bp mode on NovaSeq6000. The downstream computational analysis was made by FASTQc v0.11.9, BowTie2 v2.4.5, SAMtools v1.15.1 for checking the data quality, aligning to the reference genome and obtaining the files necessary to visualize the alignment. The alignment was firstly visualized on Integrated Genome Viewer (IGV) v2.12.3. The files generated via SAMtools were analysed using an in-house developed tool, QSA vbeta-1, obtaining position-specific entropy measures. Based on entropy, a distance matrix was computed using a script on Rstudio v2022.02.2+485.

Visualization with IGV suggested an overall small mutation rate in the samples; while the analysis QSA confirmed the trend, it gave a different insight for both inter-sample and intra-sample variability. We retrieved graphs highlighting the entropy per position, calculated using Shannon entropy. From the entropy values we retrieved the overall variability of each sample, in the range 0.08-0.35. Exporting the Position Probability Matrix from QSA to RStudio, we were able to compute a distance matrix, yielding distances based on Shannon Entropy values in the range of 205.34-5.10, allowing for a nodal graph representation of genomic diversity.

This analysis workflow and developed bioinformatic tools allowed to overcome limitations due to simple consensus sequence determination, and incorporate the in-depth sequence information obtained from NGS techniques into a unified intra-sample and inter-sample genomic diversity assessment, useful for inference on phylodinamics.

P.02 *In vitro* models for the study of B19V interaction with the human placental BeWo monolayer

Francesca Bonvicini, Gloria Bua, Erika Fasano, Elisabetta Manaresi, Giorgio Gallinella

University of Bologna – Dept of Pharmacy and Biotechnology

Parvovirus B19 (B19V) is a human pathogenic virus characterized by a selective but not exclusive tropism for erythroid progenitor cells. In pregnancy, B19V poses a potential risk to the fetus as it can spread transplacentally leading to serious fetal complications such as anemia and hydrops, cardiomegaly and pericardial effusion, spontaneous abortions and intrauterine death. Although the etiologic role of B19V in these

adverse outcomes has been widely recognized, the interaction between B19V and the human trophoblasts cells within the placenta has not yet been comprehensively explained.

We have therefore established *in vitro* experimental models based on the BeWo human choriocarcinoma cell line. BeWo cells show trophoblast-like appearance and form a confluent, polarized monolayer resembling first trimester placenta in structure and function; they can also be induced to undergo terminal differentiation and syncytialization, thus mimicking a mature placenta, when cultured in the presence of forskolin. A BeWo polarized monolayer using a collagen-coated membrane as scaffold was established, where the integrity was validated by cell morphology, biophysical features, and immunostaining of specific membrane proteins. Once obtained, these experimental models were used to detail B19V infection of human trophoblasts, and its transport across the placental barrier.

The presence of the globoside as a cellular receptor was confirmed by cytofluorimetric analysis both on the cytotrophoblasts and on the syncytiotrophoblasts and evaluated at similar levels (25.7 ±10% and 28.1 ±9.8%, respectively). No evidence of productive infection was observed in the undifferentiated and forskolin-treated BeWo cells (50 µM) since viral DNA amounts gradually decreased in cells and in the corresponding supernatants. In addition, no mRNAs and accumulation of capsid proteins were detected in the unpolarized BeWo cell monolayers along a time course of infection, up to 72h. In polarized trophoblasts, a transcytosis mechanism can occur as demonstrated by kinetics of viral DNA accumulation.

Knowledge on how B19V interacts with maternal-fetal interface will help to establish a suitable system to evaluate the effectiveness of the protective mechanisms operated by maternal antibodies against intrauterine infection, to assess the inhibitory activity of antiviral drugs and other therapeutic options that are not currently present.

P.03 Mesenchymal stem cells are susceptible but non-permissive to B19V replication

Gloria Bua[1], Pasquale Marrazzo[2], Francesco Alviano[2], Laura Bonsi[2], Giorgio Gallinella[1]

[1]Department of Pharmacy and Biotechnology, University of Bologna, Bologna, Italy. [2]Department of Experimental, Diagnostic and Specialty Medicine, University of Bologna, Bologna, Italy.

Mesenchymal stromal cells (MSCs) are multipotent stem cells with high expansion potential and self-renewal capacity. Their main characteristic is the ability to differentiate in vitro into multiple lineages, including osteocytes, chondrocytes, adipocytes and endothelial-like cells. Due to these characteristics, MSCs are good candidates for regenerative medicine and cell therapy strategies. The risk of transmitting viruses from ex vivo expanded MSCs is of particular concern, especially in immunosuppressed patients. Parvovirus B19 (B19V) is a human pathogenic virus with a specific tropism for bone marrow erythroid progenitor cells and the property to persist in several tissue types, including bone marrow. The virus can also cross the placenta and infect fetal erythroid progenitors. Thus, we investigated the susceptibility and permissiveness of bone marrow and placenta-derived MSCs to B19V to understand their potential role in the risk of B19V transmission. Mononuclear cells were isolated by Ficoll-Paque centrifugation of bone marrow blood from healthy donors and grown in DMEM containing 20% FBS at 37°C in 5% CO_2. Non-adherent cells were removed after one week and medium regularly changed. Expression of surface antigens, specific for defining the identity of MSCs, was measured by flow cytometry, as well B19V receptor moieties. For infection experiments, cells were inoculated with B19V viremic serum in order to obtain a moi of 10^4 geq/cell. After the absorption period of 2 h at 37°C, infected cells were washed and seeded at an initial density of 10^6 cell/ml. Aliquots of cells were collected at different time post infection and analyzed for the presence of viral DNA and RNA by qPCR and qRT-PCR assays. Cells expressed the standard MSC markers, such as CD29, CD44, CD73, CD90, and OCT-4 and expression was maintained after infection, while only a small fraction of cells expressed globoside. Viral DNA became associated to cells, but analysis of viral nucleic acids revealed that neither replicative nor transcriptional activity occurred. However, viral DNA persisted in late time post infection. Our results showed that MSCs are susceptible but not a permissive environment to B19V productive infection and the presence of B19V DNA did not alter the expression profile of surface antigens. Further investigations are needed to

understand the possible consequences on the cell physiology, like an impaired differentiation ability, as well the capability of virus reactivation after cell differentiation.

P.04 The role of Host Cell Factor 1 in the life cycle of AAV

Caroline Dierckx[1], Zander Claes[2], Mathieu Bollen[2], Els Henckaerts[1,3]

[1] Laboratory of Viral Cell Biology and Therapeutics, Department of Cellular and Molecular Medicine & [3] Department of Microbiology, Immunology and Transplantation, KU Leuven, Belgium; [2] Laboratory of Biosignaling and Therapeutics, Department of Cellular and Molecular Medicine, KU Leuven, Belgium

The persistent and non-pathogenic character of AAV offers great opportunities for its usage as therapeutic DNA delivery vehicle. However, uncertainties regarding long-term efficacy are surfacing in clinical trials. As transcription levels appear to go down over time in certain treatments, the question arises whether these could be due to epigenetic changes of the recombinant genome. The epigenetic regulation of the AAV genome remains largely unexplored, including the mechanisms that contribute to latency of wildtype AAV2 and the switch from latency to lytic replication. Key in this switch are the replication proteins (Rep) and their cellular interaction partners. By executing Bio-ID studies, our laboratory defined several proteins which are likely part of the Rep interactome. One such protein is Host Cell Factor 1 (HCF1), which is an important driver of the HSV life cycle. The interaction between HCF1 and the four Rep proteins was demonstrated in split-luciferase assays. Through pull-down experiments using Rep, HCF1 and binding mutants, we are currently validating the HCF1-Rep interactions.

Replication assays of wildtype AAV2 show increased transcription and replication in the lytic phase upon knock-down of HCF1. HCF1 is a scaffold protein for histone modifiers and in extension of our previous studies which demonstrated epigenetic silencing of the AAV2 chromatin during the latent phase, we explored if HCF1 could mediate its effect through interactions with the AAV genome and histone modifying proteins. We therefore optimized ChIP-qPCR experiments to assess the (in)direct binding of HCF1 on the viral DNA and to identify the histone modifications on the viral chromatin during the latent and lytic phase. We are also exploring if the observed effect on viral replication could be indirectly mediated through HCF1’s role in cell cycle regulation.

In summary, we have identified a new host factor which plays a role in the regulation of the AAV life cycle, which may exert its effect through histone modifications on the AAV genome. Future work will further delineate the mechanism and define the exact role of the Rep proteins.

P.05 Analysis of Parvovirus B19 transcriptome in UT7/EpoS1 cells by mRNAseq techniques

Erika Fasano, Gloria Bua, Stefano Amadesi, Alessandro Reggiani, Elisabetta Manaresi, Fabrizio Ferrè, Giorgio Gallinella

Department of Pharmacy and Biotechnology, University of Bologna, Bologna, Italy

Parvovirus B19 (B19V) is a human pathogenic virus responsible of a wide range of clinical manifestations. The ssDNA genome of 5.6 kb comprises a unique promoter directing the transcription of a pre-mRNA. A complex array of viral mRNAs is produced as a result of the diverse combinations of possible splicing and cleavage/polyadenylation events. The frequency of the processing events is different during infection leading to a two-state, early/late expression profile. The aim of this study is a detailed characterization of B19V transcriptome during a course of infection in UT7/EpoS1 cells through an advanced approach of Next Generation Sequencing (RNA-Seq). B19V-infected UT7/EpoS1 cells were analysed at 0, 2, 16 and 48 hpi. Viral replication and transcription were assessed by qPCR e qRT-PCR, and capsid protein expression was observed by immunofluorescence. For RNA-Seq analysis, total RNA was extracted and purified from infected cells. Sequencing of RNA samples was performed on paired-end 150 bp mode on NovaSeq6000 (Illumina), by IGA Tehcnology Services (Udine, Italy). Downstream computational analysis was performed using the

following tools: Trimmomatic 0.39, HISAT2 2.2.1 and StringTie 2.2.1 for trimming, mapping and counting the reads, respectively, and DESeq2 for the differential expression test.

We observed an increase (+ 1 Log) of the amount of viral DNA and total RNA at 48 hpi by qPCR and qRT-PCR. Using different sets of primers, we detected an earlier appearance of non-structural (NS) protein mRNAs at 16 hpi, compared to the capsid proteins mRNAs that accumulated later in the course of infection. RNA-Seq analysis showed that 0.01% of the reads were correctly mapped on reference B19V genome at 16 hpi increasing to 0.5% at 48 hpi. Bioinformatics data confirmed the prevalence of NS mRNA in the early stage of infection, while VP mRNAs were abundant in the late stage. Correct usage of cleavage polyadenylation sites and splice junctions was also confirmed. Overall, results are coherent with the two-state transcription profile, early and late, during B19V infection in UT7/EpoS1 cells, allowing a precise mapping and fine characterization of the frequency of the diverse species of B19V mRNA. In addition, viral transcriptome analysis was coupled to a deep investigation of the cellular transcriptome, highlighting a massive variation in the cellular expression profile during B19V infection.

P.06 Prevalence of Human Chaphamaparvoviruses

Jingjing Li[1,2], and Maria Söderlund-Venermo[1]

[1]Department of Virology, University of Helsinki, Helsinki, Finland; [2]Hubei Key Laboratory of Edible Wild Plants Conservation & Utilization, Hubei Engineering Research Center of Characteristic Wild Vegetable Breeding and Comprehensive Utilization Technology, Hubei Normal University, Huangshi, P.R. China

Since 2012, a group of parvoviruses belonging to a newly formed genus, *Chaphamaparvovirus* of the subfamily *Hamaparvovirinae*, have been identified by metagenomic sequencing in different vertebrates. Chapparvoviruses (ChPVs) are likely to be widespread in nature and have been detected in many species of animals including birds, fish, reptiles, and mammals as well as rhesus macaques, but as members of a novel genus, ChPVs still have some uncertainties and it is unclear whether these viruses are pathogenic. Nevertheless, a recent study showed that a novel ChPV, the mouse kidney parvovirus (MKPV), is associated with kidney tubulointerstitial fibrosis in mice.

In 2020, researchers identified the first human ChPV from plasma of a Brazilian patient with dengue-like symptoms and characterized its partial genome. These prior reports of ChPVs in numerous mammals including non-human primates make HChPV a plausible human-infecting virus.

In this study, we present a real-time quantitative PCR assay for the detection of such novel HChPVs. Differentiation of the individual HChPV species can be subsequently achieved by sequencing. This PCR is used to screen stool, nasal swab and serum or plasma samples from patients with and without gastroenteritis or respiratory-tract infections. Moreover, the VP2 gene will be cloned into a bac-to-bac plasmid and used to create recombinant VP2 virus-like particles (VLPs). Biotinylated VLPs are then used as antigen in a novel in-house IgG EIA. Serum or plasma samples are then analyzed to explore the seroprevalence of human chapparvoviruses.

P.07 Brain organoids as a platform to study subcellular trafficking of recombinant AAV vectors

Marlies Leysen[1], Idris Salmon[2], Sereina O. Sutter[3], Cornel Fraefel[3], Adrian Ranga[2], Benjamien Moeyaert[1], Els Henckaerts[1 4]

[1] Laboratory of Viral Cell Biology and Therapeutics, Department of Cellular and Molecular Medicine & [4] Department of Microbiology, Immunology and Transplantation, KU Leuven, Belgium; [2] Laboratory of Bioengineering and Morphogenesis, Biomechanics Section, Department of Mechanical Engineering, KU Leuven, Belgium; [3] Institute of Virology, University of Zurich, Switzerland

Adeno-associated virus (AAV) is the leading gene therapy vector in clinical development. However, efficacious vector delivery to target cells remains a challenge. The subcellular bottlenecks underlying

suboptimal transduction remain elusive and progress in this area may have been hampered by the lack of model systems that mimic human tissues. In this study we explore if human organoids represent a human-like setting which can be used to study the transduction process of recombinant AAV vectors. To assess the potential of this model system we use human brain organoids to investigate how the subcellular trafficking process in neurons differs between AAV True Type (AAV-TT) and its ancestral AAV2. AAV-TT harbors 14 amino acids (AAs) substitutions as compared to AAV2, which culminate in superior transduction in the central nervous system of rodents and non-human primates.

We have successfully generated human induced pluripotent stem cell-derived brain organoids, which display characteristic markers as determined by immunostaining. Importantly, we demonstrate that AAV-TT, which cannot bind heparan sulphate proteoglycan, has the ability to transduce organoids. This is in stark contrast to observations made in cell lines and 2D primary cell cultures, where only infections with AAV2 result in successful transduction. These data indicate that this model system provides similar transduction profiles as observed *in vivo*.

In preparation of vector trafficking studies in human brain organoids, we performed AAV capsid labelling studies in HeLa cells using confocal (1-photon, multi -photon) microscopy and set up an IF-FISH assay in human fibroblast cells in order to visualize viral genome release. In the next phase of the study, we will apply these techniques to track how AAV2 and AAV-TT differ in cellular uptake, trafficking, nuclear translocation and genome release in human brain organoids. If successful, these studies will lead to a better understanding of capsid sequence-structure-function relationships and allow the creation of novel rationally designed vectors fit for transduction in human tissues.

P.08 Equine Parvovirus Hepatitis qPCR screening of stored equine heparin-plasma and serum samples with and without increased liver enzyme activities

Anna Sophie Ramsauer[1], Ina Mersich[1], Irina Preining[1], Jessika-Maximiliane Cavalleri[1]

[1] Clinical Unit of Equine Internal Medicine, University Equine Clinic, University of Veterinary Medicine Vienna, Austria

Equine Parvovirus Hepatitis (EqPV-H) is associated with Theiler's disease, a potentially life-threatening fulminant hepatic necrosis. The global EqPV-H DNA-prevalence in horses without clinical signs is 7-17%. The evaluation of its association with liver abnormalities in subclinical infected horses requires further investigations. Therefore, the aim of this study was to establish an assay to screen stored heparin-plasma and serum samples of horses with and without increased liver enzyme activities for the presence of EqPV-H DNA. As heparin has an inhibitory effect on PCR, protocols to overcome this issue using heparinase digestion and an alternative mastermix were investigated. A duplex assay to screen for EqPV-H and the genomic single copy gene TTC17 simultaneously was established using a previous standard PCR protocol including mastermix (M1). Severity of inhibition in spiked serum and heparin-plasma samples was determined. A digestion step in isolated samples prior to PCR using different concentrations of *Bacteroides* Heparinase I (0.2 – 2 units per 3µl reaction) was evaluated. Additionally, the performance of an alternative mastermix (M2) was tested. Then 94 stored equine serum and heparin-plasma samples with normal (73) and increased (21) liver enzyme activities (GLDH and GGT) were preliminary screened.

Using M1 severe inhibition was present in heparin-plasma samples yielding not any CT values. Digestion of isolated DNA with one unit of *Bacteroides* Heparinase I per 3µl reaction was the lowest concentration, which completely prevented heparin induced PCR inhibition. The use of M2 significantly reduced the inhibition in plasma, resulting in a mean ΔCT of 1.2 (SD: 0.7) compared to serum, while general PCR performance was improved by a mean ΔCT of 1.4 (SD: 0.3) compared to M1. Therefore, M2 was selected for preliminary screening of 94 stored serum and heparin-plasma samples. EqPV-H DNA was detectable in 13% of horses. The prevalence was 10% (2/21) in horses with elevated liver enzyme activities and 14% (10/73) in horses with values within the reference range.

In summary useful protocols for PCR screening of heparin-plasma samples were established. No association between EqPV-H and increased liver enzyme activities could be determined by this preliminary screening. Further screenings and analyses of stored samples are ongoing.

P.09 FLIM-FRET studies of AAV capsid nuclear disintegration

Visa Ruokolainen[1], Michael Kann[2], Maija Vihinen-Ranta[1]

[1] Department of Biological and Environmental Science, University of Jyvaskyla, Finland; [2] University of Gothenburg, Gothenburg, Sweden

Adeno-associated virus (AAV) genome is released from the capsid in the nucleus. However, the exact spatial information of this event is unknown: is it in the vicinity of the nuclear envelope, or somewhere further inside the nucleoplasm?

To study the nuclear disassembly of AAV-2 capsids, we used a quantitative FRET (Förster resonance energy transfer) -based fluorescence lifetime imaging microscopy (FLIM). FLIM-FRET is a quantitative method to study very close proximity of two fluorophores. When the fluorophores are close enough, the FRET donor fluorophore lifetime decrease. When the fluorophores withdraw further from each other, the FRET loses its efficiency and the donor lifetime increase reaching finally its normal lifetime.

In this ongoing project, AAV capsids were stained with two NHS reactive fluorescent dyes, a FRET pair. Using lower and higher amount of the fluorophores, we could see 6% 12% decrease in the donor lifetime, respectively, in comparison to donor-only labelled viruses in vitro. This implies that the FRET is observed, and it is more efficient when more dye molecules are bound on the viruses. FRET was observed at least until 3 hours post infection in fixed cell samples, indicating that travel through endosomes to the perinuclear area did not affect the FRET of labelled capsids.

Before moving to live cell imaging, we are in a process to characterize the infectivity of the labelled viruses and optimize the labelling protocol for efficient FRET without compromising the infection potency.

P.10 Enhanced transduction efficiency of AAV vectors mediated by polyvinyl alcohol and human serum albumin is serotype- and cell type-specific

Jakob Shoti, Claire K. Scozzari, Reema Kashif, Hua Yang, Mengqun Tan, Wei Wang, Keyun Qing, and Arun Srivastava

University of Florida, USA

Human serum albumin (HSA) has been reported to increase the transduction efficiency of several AAV serotype vectors in human hepatocellular carcinoma cell line, Huh7 (*Gene Ther*, 24: 49-59, 2017). We have reported that polyvinyl alcohol (PVA) increases the transduction efficiency of AAV6 vectors in both human erythroleukemia cell line, K562, and in primary human hematopoietic stem cells, and that the observed increase is due to direct interaction between PVA and AAV6 capsid (*Mol Ther Nucl Acids*, 20: 451-458, 2020). In the current study, the following two questions were addressed: (i) Does PVA also increase the transduction efficiency of AAV3 serotype vectors in view of their remarkable tropism for human liver (*Hum Gene Ther*, 31: 1114-1123, 2020); and (ii) Do PVA and HSA have an additive effect on the transduction efficiencies of AAV3 as well as AAV6 vectors given their high-efficiency transduction of primary human hematopoietic stem cells (*Sci Rep*, 6: 35495, 2016). Transduction efficiencies of AAV2, AAV3, and AAV6 vectors, with and without preincubation with PVA, HSA, or PVA+HSA, were evaluated in HeLa, Huh7, and K562 cells, respectively. PVA failed to increase the transduction efficiency of AAV2 vectors, and no increase with HSA alone, or PVA+HSA together was observed in HeLa cells. The transduction efficiency of AAV3 vectors was significantly increased by both PVA and HSA, but no additive effect was observed in Huh7 cells. Similarly, no additive effect was seen in the transduction efficiency of AAV6 vectors in K562 cells. Human HepG2 cells, known to express and secrete human clotting factor IX (hF.IX) were either mock-transduced or transduced

with scAAV3-hF.IX vectors, with and without preincubation with HSA or PVA. HSA had no effect, but preincubation with PVA led to ~5-fold increase in mock-adjusted hF.IX protein expression without PVA in HepG2 cells. These data suggest that PVA/HSA-AAV interactions occur by different mechanisms, and that enhanced transduction efficiency of AAV vectors mediated by PVA and HSA is serotype- and cell type-specific. The use of PVA is an attractive strategy to further improve the efficacy of clinically relevant AAV serotype vectors for their optimal use in human gene therapy.

P.11 Development of allele-specific dual PCRs to identify members of the 27a cluster of PPV1

Vivien Tamás, Ferenc Olasz, István Mészáros, István Kiss, Zalán G. Homonnay, Preben Mortensen and Zoltán Zádori

Veterinary Medical Research Institute, Budapest, Hungary

The Porcine Parvovirus 1 (PPV1) is considered one of the major causes of severe reproductive failure in swine. The clinical signs of PPV1 infection can only be observed in pregnant pigs, it causes the SMEDI (stillbirth, mummification, embryonic death, and infertility) syndrome. Until the 2000s, PPV1 was thought to be very stable immunologically therefore genetic changes of the viral genome were not studied systematically. Recently a novel genotype was identified in Europe and the PPV1-27a was named as the prototype of new genetic cluster. It has been suggested that members of the PPV1-27a cluster may reduce the efficacy of currently available vaccines. The aim of our work was to update the definition of 27a, therefore we aligned 93 databank-deposited partial or full nucleotide and protein sequences of the VP2 of different PPV1 isolates. It was confirmed that the 27a cluster could indeed be distinguished from other members of the other PPV1 strains. However, some divergences were identified compared to earlier defined genetic markers. Based on the genetic differences we developed a dual allele-specific PCR for easy and quick discrimination of members of the 27a cluster from other PPV1 strains. The detection limit of dual PCR was found <1.66 x10^4 copies/reaction. To sensitize and make it more user friendly, the method was further developed for qPCR application, with fluorescent probes. Regarding the detection limit of the two PCRs (<1.66 x10^4 copies/reaction of the dual PCR versus <2.40 x10^2 copy/reaction of the dual qPCR) approximately two log improvement was achieved in the sensitivity of the method.

P.12 A record of Parvovirus B19 laboratory diagnosis in endemic/epidemic or COVID pandemic

Simona Venturoli[1]; Alessia Bertoldi[1]; Elisabetta Manaresi[2] and Giorgio Gallinella[1,2]

[1]Microbiology Unit, IRCCS Azienda Ospedaliero-Universitaria di Bologna, Italy. [2]University of Bologna – Dept of Pharmacy and Biotechnology

Background and Objectives: Parvovirus B19 (B19V) is a widespread human virus, normally transmitted through the respiratory route. The aim of our study was the survey of diagnostic activity for detection of antibodies to B19V and viral DNA, carried out in a metropolitan area laboratory, in the endemic/epidemic period, until 2019, or afterwards in the COVID-pandemic period.

Materials and Methods: From 2014 to 2021, 15805 consecutive serum samples were tested for the presence of parvovirus B19 IgG/IgM antibodies and 2183 serum samples were tested for the presence of parvovirus B19V DNA. Serological (indirect enzyme immunoassay test for detection of B19V specific IgM/IgG) and molecular quantitative (B19V DNA real time PCR) methods were used.

Results: IgG antibodies were detected in 8369/15805 samples (53%), IgM antibodies were detected in 487 (3.1%) and 325/2183 (14.9%) serum samples presented B19V DNA. Of 1033 sample with antibodies and molecular data, in 1.1% of cases B19V DNA load was accompanied by the presence of anti-B19V IgG, in 10% of cases B19V DNA load was accompanied by the presence of anti-B19V IgM and in 39.9% of cases B19V DNA load was accompanied by the presence of anti-B19V IgM and IgG.

A comparable IgG seroprevalence was observed in endemic/epidemic (aa 2014-2019; 53%) and COVID pandemic (aa 2020-2021; 54%). Instead we observed a significative difference in IgM seroprevalence (3.1% vs 0.7%) and in viral DNA detection among endemic/epidemic (B19V DNA: 20.0%) and COVID pandemic (B19V DNA 9.4%). In particular, 2019 showed the highest B19V incidence (5.02% IgM, 21.7% B19V DNA) and the lowest incidences occur in 2021(0.59% IgM, 8.01% B19V DNA).

Conclusions: During the SARS-CoV-2 pandemic, several studies reported a lower incidence of respiratory infections other than COVID compared to previous years. This survey of B19V laboratory diagnosis indicates that only a small number of individuals showed exposure to B19V since the onset of COVID pandemic. Our findings confirm that public health measures taken to limit the spread of SARS-CoV-2 led to a drastic reduction of the virus circulation.

P.13 Enhanced muscle transduction by clade F vector AAVhu.32 translates across multiple animal models

Samantha A. Yost, Randolph Qian, April R. Giles, Sungyeon Cho, Chunping Qiao, Olivier Danos, Ye Liu, and Andrew C. Mercer

REGENXBIO, Inc. Rockville, Maryland 20850

We have identified AAVhu.32 as a potentially superior clade F capsid candidate for muscle-specific disease indications in a library study of 118 unique AAV capsids (A. Giles ASGCT 2021). Here we sought to further characterize this capsid in the context of a pool of 44 barcoded vectors administered intravenously into cynomolgus macaques (n=3) at $2x10^{13}$ genome copies per kg. Treated animal tissues were harvested three weeks post-injection and nucleic acids were extracted. Genome copies (DNA) and transcript copies (RNA/cDNA) were quantified via ddPCR and subjected to next generation sequencing. Compared to its closest sequence relative AAV9, AAVhu.32 transcripts were significantly higher by double in gastrocnemius ($P < 0.0001$) and bicep ($P = 0.03$) tissue. In heart, AAVhu.32 also produced two-fold more transcript copies than AAV9, but it was not statistically significant, likely due to animal-to-animal variability ($P = 0.36$). AAV9 and AAVhu.32 transduced quadricep and tibialis anterior similarly. As hepatotoxicity is a concern in gene therapy, it is significant to note that AAVhu.32 transduced the liver at half the level of AAV9 and produced one third of RNA transcript copies compared to AAV9 ($P = 0.03$). We additionally evaluated AAVhu.32 versus AAV9 in a single vector administration study in a mouse model of Duchenne muscular dystrophy (mdx, n=5) and recapitulated results seen in the pooled vector NHP study. Most of the amino acid differences between AAVhu.32 and AAV9 lie in the VP1 unique (VP1u) region, potentially adjacent to the PLA2 domain; however, the enzymatic activity of recombinantly expressed AAVhu.32 VP1u was found to be equivalent to that of AAV9 VP1u. Rational engineering of AAVhu.32 was performed to further decrease liver transduction and boost muscle tropism through point mutations and peptide insertions. These 21 designer capsids were then evaluated in a pooled vector administration study in both wild type and mdx mice.
